# Environmental and health risks of perovskite solar modules: Case for better test standards and risk mitigation solutions

**DOI:** 10.1016/j.isci.2022.105807

**Published:** 2022-12-15

**Authors:** Christa E. Torrence, Cara S. Libby, Wanyi Nie, Joshua S. Stein

**Affiliations:** 1 Sandia National Laboratories, Albuquerque, NM 87123, USA; 2 Electric Power Research Institute, Palo Alto, CA 94304, USA; 3 Los Alamos National Laboratory, Los Alamos, NM 87545, USA

**Keywords:** Applied sciences, Environmental toxicology, Toxicology

## Abstract

Perovskite solar cells (PSCs) promise high efficiencies and low manufacturing costs. Most formulations, however, contain lead, which raises health and environmental concerns. In this review, we use a risk assessment approach to identify and evaluate the technology risks to the environment and human health. We analyze the risks by following the technology from production to transportation to installation to disposal and examine existing environmental and safety regulations in each context. We review published data from leaching and air emissions testing and highlight gaps in current knowledge and a need for more standardization. Methods to avoid lead release through introduction of absorbing materials or use of alternative PSC formulations are reviewed. We conclude with the recommendation to develop recycling programs for PSCs and further standardized testing to understand risks related to leaching and fires.

## Graphical abstract

**Figure:**
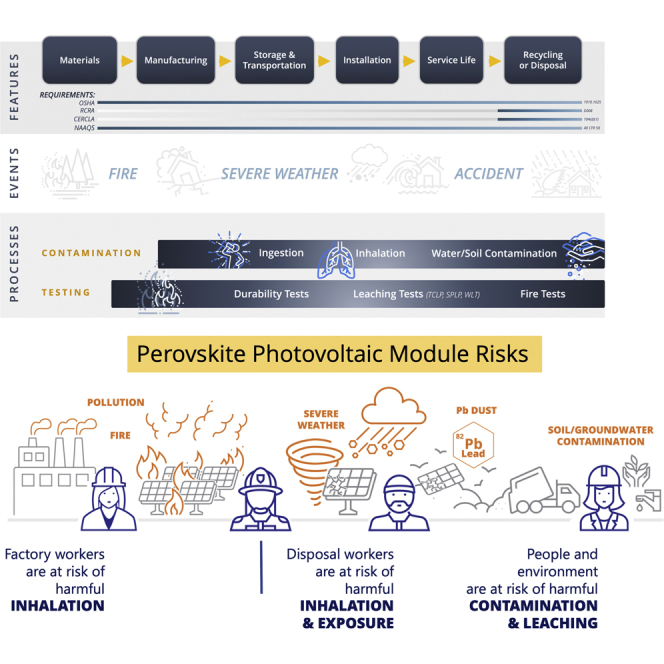

## Introduction

Metal halide perovskite solar cells (PSC), developed in the past decade, are a promising renewable energy technology due to their proven high efficiency and potential for very low production costs, such as solution processing and vapor deposition.^1^^,^^2^ However, the highest efficiency PSCs contain a soluble form of lead, which raises concerns about environmental and health risks when this technology is commercialized on a large scale. Although alternative metals are being considered, none of these formulations perform as well as lead-based PSCs. In this work, a compilation of published leaching data is presented, showing that on average the lead leaching from a broken PSC module may exceed safe limits, unless a lead sequestration layer is included in the module.

**Figure 1:**
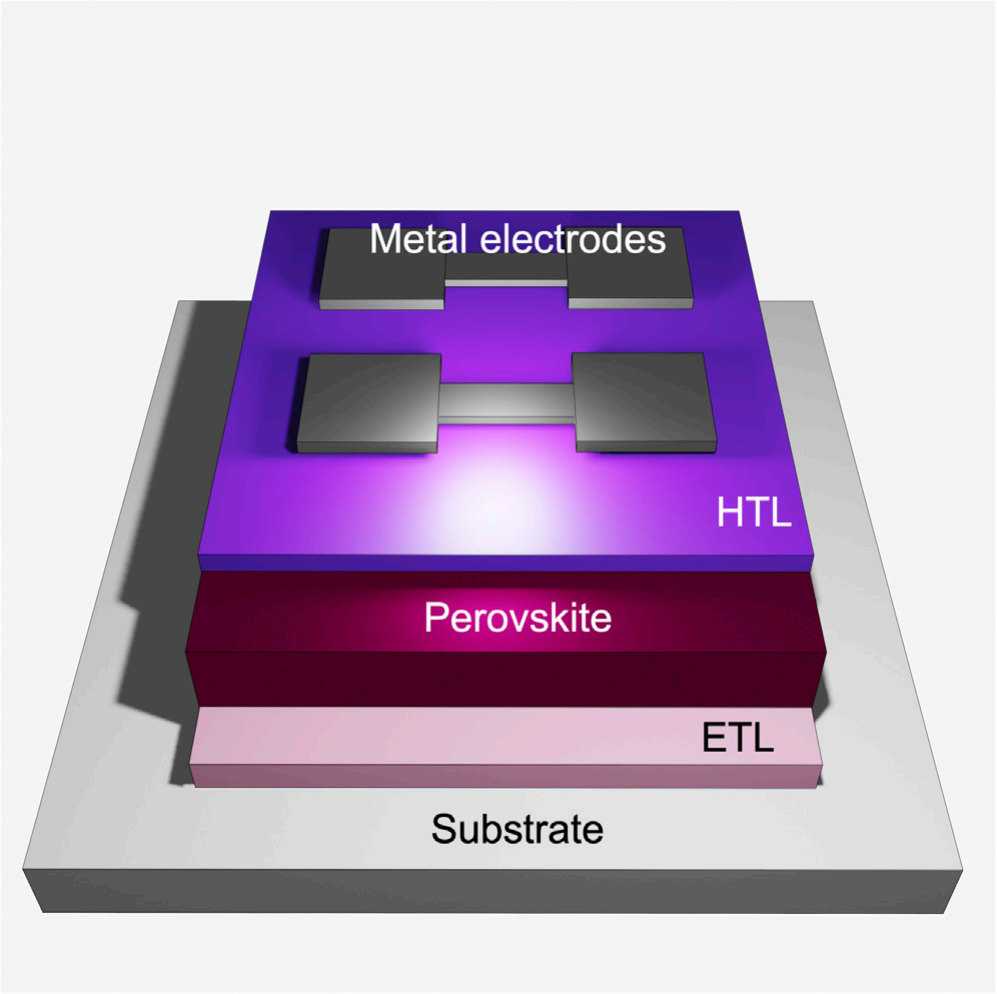
Layered components of an n-i-p perovskite PV solar cell deposited onto a transparent conductive oxide (TCO)-coated substrate, where HTL and ETL represent the hole and electron transport layers, respectively

Perovskite materials have a distinct crystallographic structure and the formula ABX_3_. For PSCs, *A* can be either an organic cation group, most commonly methylammonium (CH_3_NH_3_)^+^ (MA) and formamidinium (CH(NH_2_)_2_)^+^ (FA), or the metal Cs. *B* is a cation with a smaller radius than *A*, generally chosen to be Pb^2+^ or Sn^2+^. The anion, *X*, is a halogen such as I^−^, Br^−^, or Cl^−^. Many metal halide perovskite solar cells use blended *A*, *B*, and/or *X* sites, such as FA_0.75_MA_0.25_SnI_3_ or MASn_0.5_Pb_0.5_I_3_. Figure 1 shows the layers that compose an n-i-p PSC for reference to general cell construction.

In 2009, Miyasaka et al. reported developing a metal halide PSC with a 3.8% power conversion efficiency (PCE) using a methylammonium lead iodide (MAPI) formulation.^3^ Since then, PSCs have improved at a rapid pace. At the end of 2021, formamidinium lead iodide (FAPI), CH(NH_2_)_2_PbI_3_, and PSCs reached a PCE of 25.8%.^4^ Perovskite-silicon tandem solar cells are demonstrating record efficiencies as high as 29.5%.^5^ These values are expected to keep increasing. In addition, perovskite PV can be made using fast and low-cost solution processing and vapor deposition methods.^2^ An analysis of perovskite module manufacturing estimates single-junction perovskite modules could cost as little as $38.69 USD/m^2^.^6^ In comparison, c-Si modules currently cost between $62.90 and $79.31 USD/m^2^.^6^ The combination of high efficiency and low cost would make perovskite PV modules very competitive as long as there are no serious performance, reliability, and environmental and health risks.

## Risk assessment

To assess the environmental and health risks of perovskite PV, we follow this technology from its production to its final disposition or disposal and examine materials, processes, and unplanned events that can affect its safety. We review available data and studies that have attempted to quantify such risks.

**Figure 2:**
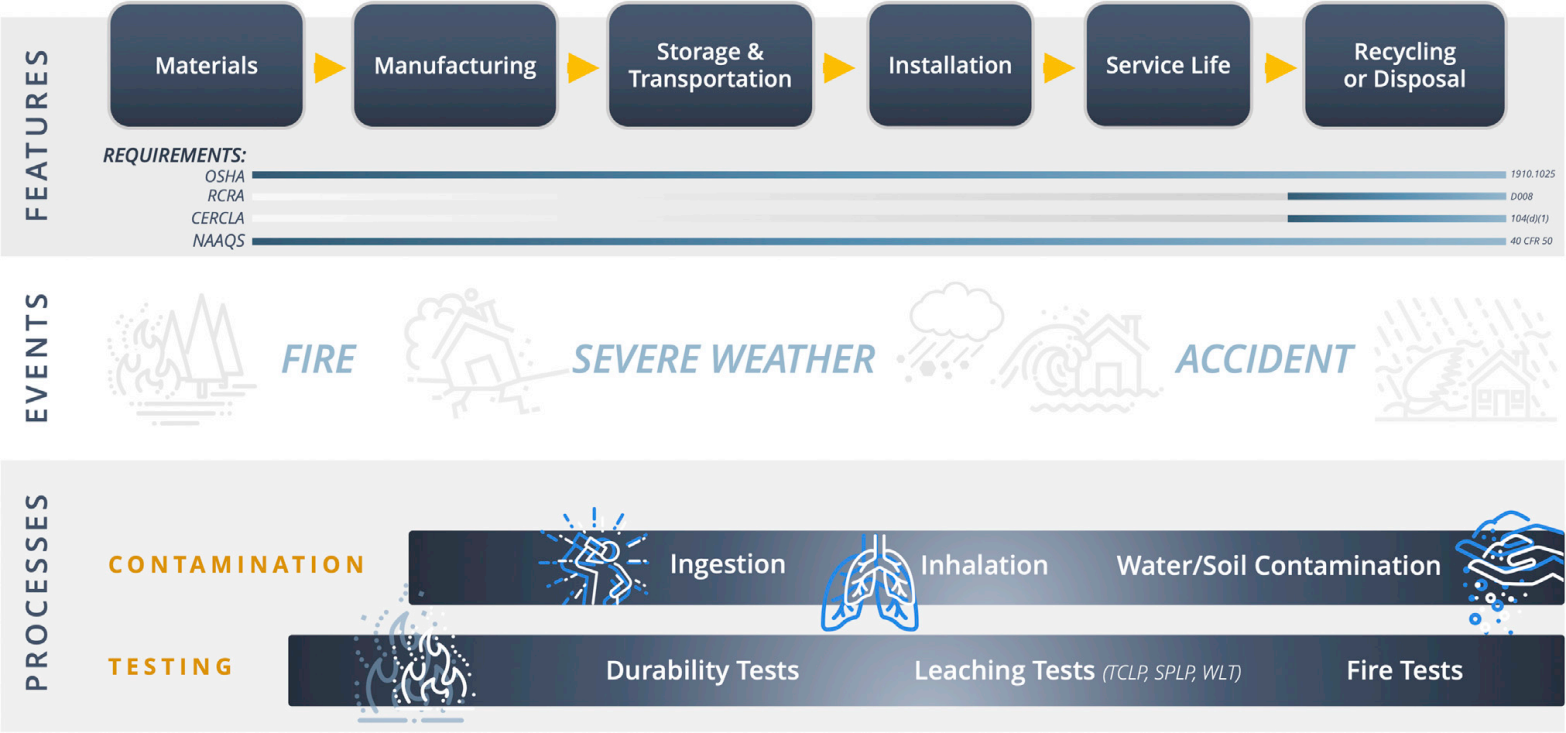
Environmental and health risks from perovskite PV technology at varying stages of the technology’s lifetime, including applicable regulations and testing methods

Figure 2 illustrates many of the features, events, and processes that affect the risk of this technology in the solar energy sector. Risk is measured as the product of the probability of exposure and its consequence. Probability of exposure is based on both the amount and concentration of toxic components as well as their mobility in the environment. It is common for risks to be low under normal conditions but to rise in certain unexpected scenarios (e.g., module breakage, fire, etc.).

### Toxicity concerns for perovskites

The bankability and commercialization of perovskite PV depends on a clear understanding of potential environmental and health risks and the ability to manage them. Lead, although harmful in any form, exists in a variety of valence states and solid forms that pose varying levels of risk due to differences in solubility and reaction products. Perovskite modules will likely contain around 0.4 g/m^2^ of lead in the perovskite layer, assuming an absorber layer thickness of 300 nm.^7^ If all of this lead is released into the top 1 cm of typical soil, the concentration would be increased by about 70 ppm. For comparison, natural lead levels in soils range from 30 to 200 ppm depending on location.^8^

MAPI formulation has several degradation pathways,^9^^,^^10^^,^^11^ reacting with water to form lead iodide:

$${CH}_{3}{NH}_{3}{PbI}_{3}\left(s\right)\overset{H_{2}O}{\to }{CH}_{3}{NH}_{3}^{+}\left(aq\right)+I^{-}\left(aq\right)+{PbI}_{2}\left(s\right)$$

At elevated temperatures, the breakdown of MAPI produces either hydroiodic acid and deprotonated MA or ammonia and methyl iodide:

$${CH}_{3}{NH}_{3}{PbI}_{3}\left(s\right)\;\Leftrightarrow {CH}_{3}{NH}_{2}\left(g\right)+HI\left(g\right)+{PbI}_{2}\left(s\right)$$

$${CH}_{3}{NH}_{3}{PbI}_{3}\left(s\right)\overset{\Delta }{\to }{CH}_{3}I\;\left(g\right)+{NH}_{3}\left(g\right)+{PbI}_{2}\left(s\right)$$

The production of PbI_2_ in all pathways is a cause for concern. PbI_2_ has a solubility constant (K_sp_) in the range of 8.3 ∗ 10^−9^ to 1.84 ∗ 10^−8^.^12^^,^^13^^,^^14^ In the presence of high temperatures, such as fire, the PbI_2_ oxidizes into PbO and PbO_2_, both of which are insoluble in water.^15^^,^^16^ The insolubility does not reduce exposure risk, however, as PbO powder can be absorbed by human skin.

Lead is of greatest concern as it is a highly toxic substance. Once inside the body, it is stored in soft tissues and, after continual exposure, is eventually absorbed into the bone and teeth, where it has an elimination half-life of 20–30 years.^17^ The US CDC sets upper limits on acceptable Pb blood level, which in the 1960s was set to 60 μg/dL and has been progressively reduced over the years to the present value of 5 μg/dL in adults and 3.5 μg/dL for children.^18^^,^^19^ In adults, even a level of 5 μg/dL can lead to anemia and increased blood pressure, and in pregnant women lead exposure endangers the fetus.^20^ For adults, symptoms of lead poisoning generally become noticeable when blood levels reach 40 μg/dL.^21^ Lead is especially dangerous to children because they absorb lead from the same source about 4–5x more than adults, and young children have hand-to-mouth tendencies.^22^ Blood levels of 5–10 μg/dL in children have been shown to cause interference with brain development, lowered IQ, decreased hearing, and stunted growth—with the impacts amplified as blood levels rise.^20^^,^^22^^,^^23^ The Institute for Health Metrics and Evaluation (IHME) estimates that as of 2019, 62.5% of the world’s intellectual disability without an otherwise obvious source is caused by lead.^24^

However, it is noted that the main hazard of lead from perovskite is the form of the lead ion leaking to the environment. As mentioned earlier, the commonly used perovskite materials in high-performance photovoltaic devices are water soluble, releasing lead iodide, which is also water soluble. On the other hand, Benmessaoud et al have dissolved PSCs in natural waters, and found that other forms of lead compounds such as lead hydroxide, lead carbonate, or lead phosphate can form^25^ because the PSCs contain anions that can react with Pb^2+^ dissolved from the perovskites. This study suggests that the Pb^2+^ may be more hazardous in biologic systems. This brings the question of the potential bio-availability of perovskite to human or other bio-systems. Patsiou et al conducted a toxicity study by exposing perovskites to zebrafish. The conclusion was that the main toxicity of the perovskites was from the bio-availability of the Pb^2+^ ions dissolved in water.^26^ This topic deserves more study.

In 2021, the World Health Organization (WHO) published a compendium of health and environmental guidance that provided 500 action items aimed at reducing disease and death caused by environmental factors. A subsection on lead risk mitigations contains 12 recommendations that include improving standards for manufacturing and recycling lead-containing waste.^27^ July 2021 marked a milestone in the push to reduce lead exposure with the world’s last sale of leaded gasoline for cars. Leaded paint, however, is still in use, and only 41% of countries have banned leaded paint products.^24^

Tin is used in some perovskite formulations, often presented as a lead-free alternative. Although the human health risks associated with exposure to tin appear to be less harmful than those associated with lead, it should not be considered a safe and non-toxic alternative. Tin toxicity is highly dependent on its form. Inorganic tin compounds are expelled from the body quickly but have been found to be harmful in large amounts, whereas organic tin compounds, referred to as organotins, that are used in tin halide perovskites, can cause a variety of negative health effects including neurological problems, gastrointestinal symptoms, and respiratory irritation.^28^^,^^29^ Tin halide perovskites degrade to form SnO_2_ and SnI_4_.^30^^,^^31^ SnI_4_ is highly reactive with water forming SnO_2_,^30^ which can cause respiratory irritation and harm to aquatic life.^32^

Cesium has also been used to make PSCs. It is used in industrial applications in the form of cesium hydroxide. This compound can cause burns and severe irritation to the skin upon contact due to its corrosivity.^33^ Inhalation can result in irritation to the nose, throat, and lungs, potentially leading to a buildup of fluid in the lungs.^34^ The National Institute for Occupational Safety and Health (NIOSH) recommends that workers not be exposed to more than 2 mg/m^3^ of airborne cesium hydroxide.^34^ Cesium hydroxide also poses a risk to workers in transport and manufacturing due to its intense reactivity with water, posing a risk of burns, inhalation, and ignition of other nearby flammable materials.^33^

In addition, chromium (Cr) is found in certain PSC formulations. The toxicity of chromium is highly dependent on its ionic form. Cr^3+^ is poorly absorbed by the body, whereas Cr^4+^ is readily absorbed. Cr^4+^ inhalation causes a variety of respiratory effects, including asthma, chronic irritation, polyps, and increased risk of lung cancer.^35^ Oral exposure can result in severe liver damage and gastrointestinal hemorrhaging.^35^

An important advantage of PSCs is their ability to be made with low-cost solution processing methods. Solvents play a crucial role in the composition of the precursor solution, and the properties of the solvent dictate the crystallization nucleation and growth process, directly impacting the quality of the resulting perovskite film. As a result, solvent engineering is critical in the scaling up of perovskite module manufacturing. The most commonly used solvents include N,N-dimethylformamide (DMF), which is easily absorbed through the skin, as well as dimethyl sulfoxide (DMSO) and N-methyl-2-pyrrolidone (NMP), which are both carcinogenic and reprotoxic.^36^^,^^37^^,^^38^ Chloroform is also used in some processes, which causes severe central nervous system and liver toxicity effects.^39^ An active area of research is the use of greener solvents.^40^ Gardner et al. (2016) tested a variety of nontoxic solvents, leading to the development of a 15.1% efficient, pinhole-free PSC made with γ-butyrolactone (GBL), ethanol, and acetic acid.^41^ This device is certainly less efficient than traditionally processed perovskite cells, but it is a promising step toward the removal of toxic solvents such as DMF and DMSO. However, γ-butyrolactone is considered a restricted chemical in several countries because of its potential to be abused as a drug.^42^ Countries such as China, Poland, and Russia have restricted the use of GBL. Australia, Canada, Germany, and the US consider GBL as a controlled chemical.

### Events leading to exposure

**Figure 3:**
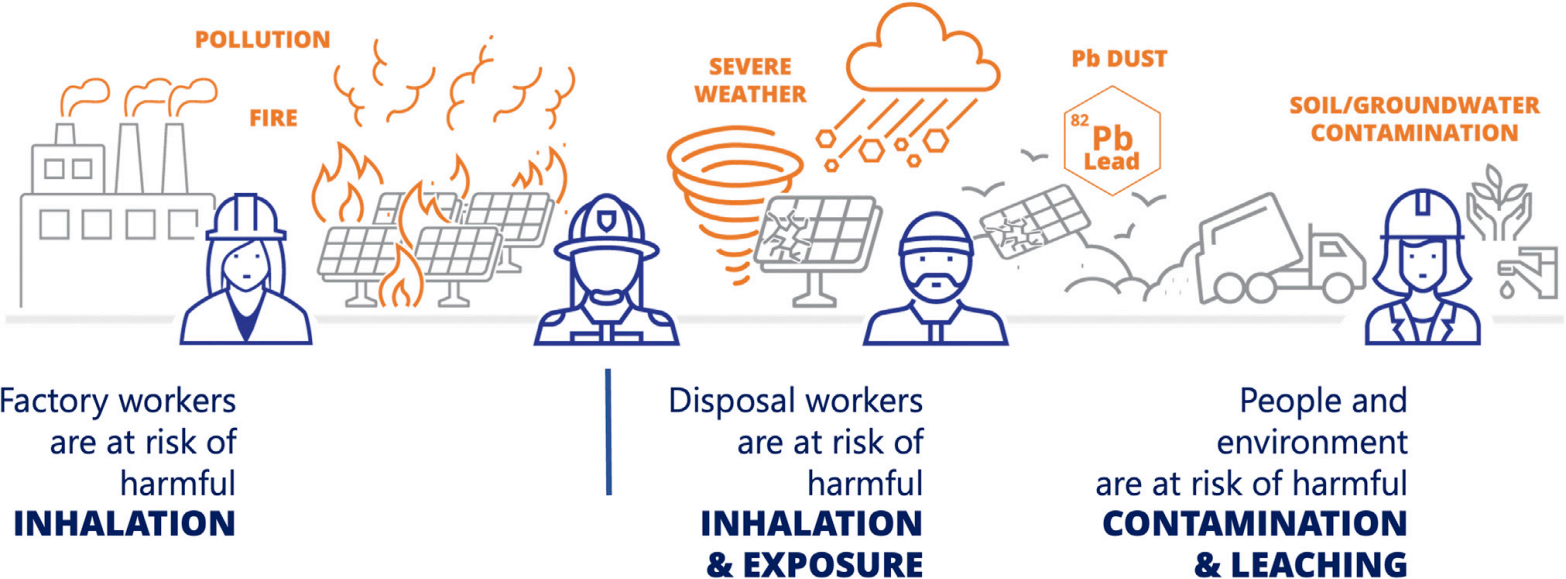
Events that potentially put workers and community members at risk of exposure to lead and other potentially harmful substances

Events that can result in exposure include accidents, disposal, and severe weather, among others. Figure 3 illustrates the types of events that could potentially endanger workers, community members, and the environment.

Accidents during manufacturing may result in exposure. Solution processing methods utilize toxic solvents. Accidental spills, inadequate ventilation, lack of safety protocols, improper hygiene and decontamination procedures, flammability risk, and non-compliance with protocols can lead to skin contact, inhalation, or even ingestion of harmful solvents.^41^ Careful precautions and protocols must be in place to protect workers during the manufacturing process.

Storage and transportation of modules also presents risks. Warehouses storing modules pose a risk in the case of a fire due to the high density of material. Transporting modules involves the risk of accident, causing module breakage and the potential release of hazardous materials, posing a skin contact risk for workers handling the broken modules. Perovskite modules are expected to have similar construction to other thin-film technologies, e.g., glass-glass modules with a polymer encapsulant. As such, data from similarly constructed modules can provide insights on breakage expectations for perovskite modules. Data from First Solar, a manufacturer of thin-film CdTe modules, show that as of 2011 approximately one-third of warranty returns due to module breakage occurred during the shipping and installation period, corresponding to the return of 0.4% of modules.^43^ Breakage, as defined by First Solar, includes chipping and other damage that would not contribute to exposing the photovoltaic layers, thus the true percentage of modules at risk of leaching or releasing hazardous material may be lower. Following installation, an average of 0.04% of modules each year are returned under warranty due to breakage or damage.^43^ In larger PV power plants, the small percentage of modules that break during installation equates to thousands of modules that need to be handled safely to avoid risk to workers. Each step in the transportation, packing and unpacking, storage, and disposal of the damaged modules all must be well controlled to minimize risks.

It is noted that the panel return rate data from CdTe modules largely depend on the packaging materials and encapsulation design employed for the specific modules. Perovskite modules are emerging technology, and the specific packaging they will adopt is uncertain. Currently, some companies are pursuing designs similar to current CdTe modules (glass-glass with encapsulant, with or without an edge seal) while others are developing flexible polymer-based packaging.^44^ The various packaging materials are nicely summarized in other review articles, and more strategies are being vigorously investigated in the field.^45^^,^^46^ The CdTe return rate data described earlier thus provide a rough guideline of the panels using the industrial standard packaging methods.

Module damage from severe weather (e.g., hail, tornadoes, hurricanes, etc.) and accidents (e.g., vehicle collisions, rocks kicked up by mowers, etc.) can lead to exposure. Communities can be impacted by lead leaching from damaged modules, contaminating nearby groundwater and soil. In addition, workers handling broken modules may be at risk of skin contact with lead-containing residue. Testing to quantify these risks is important for understanding and mitigating risks after module damage.

Landfill leaching and soil and groundwater contamination are risk factors for PSC technology at the end of life. The waste classification of PV modules determines how modules are disposed under US federal regulations, treated either in a non-hazardous (Subtitle D) landfill or in a hazardous waste facility (Subtitle C), and the associated cost. Recycling for PV modules is feasible independent of whether modules are characterized as hazardous waste. Recycling programs, technologies, and infrastructure are in their infancy, but approximately 20 US recyclers now advertise that they accept PV modules and provide other end-of-life services. This work focuses on the United States, but information about other countries and regions can be found in other reviews.^47^^,^^48^

### Exposure pathways

There are three main exposure pathways of concern: inhalation, ingestion, and skin contact. All of these pathways require that the contaminants are exposed to the environment and are mobile.

Inhalation risk stems primarily from fire smoke, but toxic substances in dust or other small particles can be aerosolized from burned or broken modules and cause resultant harm. For some toxic substances, inhalation is the highest risk exposure pathway due to increased absorption by the body. For example, almost all inhaled lead is absorbed by the body for both adults and children.^22^ Solvents used in the production process pose inhalation risk, as they are also highly volatile. Common safety practices include maintaining proper ventilation, wearing well-fitting masks with suitable filters, and restricting non-essential workers from accessing areas with solvents.^49^ The lack of data on exposure risks from perovskite modules in fires is an important research gap.

Ingestion can occur through several routes, including contamination of drinking water, children’s play areas, or soil used to grow food. Furthermore, improper adherence to hygiene protocols can lead to accidental ingestion of toxic materials. For example, if proper glove wearing and/or handwashing protocols are not followed, workers can contaminate their personal belongings and food.^50^

An additional route of lead ingestion is the consumption of plants grown in lead-contaminated soils. Research has shown that plants bioaccumulate lead through their roots when planted in Pb-contaminated soils, as well as foliar (leaf) absorption from contact with lead oxide nanoparticles.^51^^,^^52^ Co-locating agriculture and livestock grazing with solar farms (i.e., agrivoltaics) is an emerging trend, meaning any contamination of the soil within the power generation facility where solar modules are installed while in operation could pose food chain risks. Li et al. (2020) showcased the impact of increased Pb uptake in mint plants exposed to contaminated soil.^51^ A key takeaway from this study is that the lead absorption was higher from lead-halide-perovskite-contaminated soils than lead-iodide-contaminated soils, indicating the solubility of lead halide perovskites poses a higher risk.^51^

No data are currently available on the dermal absorption of perovskite dust that can become airborne during the handling of damaged modules or if modules are crushed during recycling or disposal, highlighting an important knowledge gap. The degradation products of perovskites can be assessed, however. Filon et al. (2006) demonstrated the absorption of PbO through the skin with the important finding that the use of liquid soap to remove the PbO powder actually increased skin absorption of PbO.^53^ These data highlight the importance of preventing skin contact with lead oxide material, as well as the need for education and effective cleansing methods.

Skin contact is an important exposure pathway for liquid solvents, which can cause skin irritation and burns. Some chemicals can also be absorbed into the body through the skin, putting one at risk for both dermal and internal harm. Knowledge of proper personal protection equipment (PPE), particularly gloves, is crucial to prevent skin contact. For example, DMF is a commonly used solvent that is readily absorbed through the skin and can act as a transport agent to other substances. Neither latex nor nitrile gloves offer adequate protection from the solvent, meaning manufacturers need to ensure that their workers have the proper PPE, such as gloves impenetrable by the solvents.

### Regulatory environment

The US Department of Labor’s Occupational Safety and Health Administration (OSHA) enforces safety regulations to provide safe and healthful working conditions for workers.^54^ OSHA regulates a wide range of workplace hazards, such as fall risk, chemical hazards, and safe use of equipment.^54^ This includes the assembly of modules as well as the production of each component. Glass production, for example, is heavily regulated to protect workers from silica dust and exposure to high temperatures.^55^^,^^56^ OSHA also protects workers across the lifetime of a PV module, including installation, shipping, and disposal of modules.^54^

An estimated 1.6 million workers in the US are potentially exposed to lead, primarily at construction sites and manufacturing facilities. OSHA standard 1910 regulates a wide range of worker safety issues, including lead exposure, by defining the maximum worker exposure of 50 μg/m^3^ of lead in the air, averaged over an 8-hour period.^57^ Employers must take compliance actions if airborne lead levels exceed the “action limit” of 30 μm/m^3^, including medical monitoring; more stringent worksite controls; and the use of respirators, gloves, goggles, and other PPE.^57^ In addition, if lead dust or lead-containing materials are present in a workspace, employers must provide proper PPE and require good hygiene practices, such as regular hand washing and showering before leaving the workspace.

The National Ambient Air Quality Standards (NAAQS) regulates the ambient air concentration of lead, ozone, carbon monoxide, particulate matter, sulfur dioxide, and nitrogen dioxide. The maximum safe lead level in ambient air is defined as 0.15 μg/m^3^ over a three-month average.^58^

Solid waste in the United States is regulated under the Resource Conservation and Recovery Act (RCRA).^59^ This regulation aims to minimize the disposal risks of hazardous substances by regulating the generation, transportation, treatment, and disposal of hazardous waste. Lead is a highly regulated pollutant, but exposure to other heavy metals and silica is controlled as well.

Per the US Environmental Protection Agency (EPA), PV modules are categorized as solid waste and are required to undergo waste characterization before disposal. The EPA specifies the Toxicity Characteristic Leaching Procedure (TCLP) test (Method 1311) under RCRA for waste characterization.^60^ The procedure simulates leaching in a landfill to determine whether hazardous elements will leach from the waste. There are currently TCLP limits on eight heavy metals, some of which may be present in perovskite solar modules, as shown in Table 1. Any material with TCLP analytical results above the regulatory limit is deemed hazardous waste and must be disposed of in facilities permitted to accept such waste. In addition, hazardous waste is subject to Department of Transportation regulations, adding complexity and cost to the transportation of modules at the end of their lifetime.^61^

**Table 1:** RCRA heavy metals concentration limits

Analyte	Limit (mg/L)
Arsenic	5
Barium	100
Cadmium	1
Chromium	5
Lead	5
Mercury	0.2
Selenium	1
Silver	5

The Comprehensive Environmental Response, Compensation, and Liability Act (CERCLA) of 1980 focuses on the response to a release, or the threatened release, of hazardous substances that may endanger public health or the environment.^62^ A site containing high levels of lead in the soil or groundwater meets criteria to be deemed a “superfund site” and qualify for funding to clean up and rehabilitate the site. The risk of lead at such sites is evaluated using models such as the Integrated Uptake Biokinetic (IEUBK) model and the Adult Lead Methodology (ALM).^63^ CERCLA assesses potential superfund sites for a variety of contaminants, and lead is the most common pollutant. There are currently over 1,212 recorded superfund sites with lead identified as a contaminant of concern. EPA guidance indicates that levels of 400 mg/kg and 800 mg/kg may be appropriate screening values for soils at residential and commercial/industrial sites, respectively. EPA notes that potential combined exposure to soils with lead at these screening levels and lead from other sources, such as drinking water, could result in exceedances of threshold blood-lead levels.

Several states in the US are taking independent action to regulate PV module disposal. Washington state has an active law requiring PV manufacturers to finance the takeback and recycling of modules sold in the state.^64^ In 2021, California’s rule to consider PV modules as universal hazardous waste took effect, allowing for less stringent storage and transportation requirements for end-of-life modules.^65^ Spent modules designated as universal waste may be accumulated for a year instead of 90 days, enabling bulk shipment to a recycling facility. Also, there are fewer inspection, recordkeeping, and labeling requirements for universal waste, and a hazardous waste manifest is not required for shipping. New Jersey and North Carolina have each funded studies on the disposal of modules to review options for their respective legislation on the matter. North Carolina’s study recommended the state pursue classifying PV modules as universal waste, relieving the burden of the hazardous waste categorization.^66^ New Jersey’s investigation is still underway.

## Methods of estimating environmental risks

### Lead leakage caused by severe weather conditions

Severe weather events with high winds, such as hurricanes and tornadoes, can damage PV power plants and modules but the level of damage depends on site and storm specific features. The Category 5 Hurricane Maria damaged only 0.002% of modules in a Puerto Rican solar farm. Category 4 Hurricane Florence damaged 0.52% of modules in a solar farm in North Carolina. In 2015, a tornado struck Desert Sunlight Solar Farm in southern California, home to 8.8 million modules. Approximately 1.8% of the modules were damaged.^67^ A 2019 hailstorm at another North Carolina solar site initially was thought to have caused damage to 31% of modules based on visual inspection, but subsequent UV fluorescence imaging determined that 51.5% of modules had broken glass and/or internal cell cracking.^68^ An important risk that needs to be evaluated is how long broken modules remain in the field before they are discovered, collected, and disposed; the likelihood of leaching during subsequent environmental exposure; and whether there is a risk of soil and groundwater contamination.

Chen et al. (2020) tested damaged perovskite minimodules outdoors during a simulated rainstorm that resulted in 16.7 mm of rain, collecting the rainwater runoff from each module. The data revealed that the runoff had a lead concentration of 0.432 mg/L.^69^ This value is noticeably lower than the 15.5 mg/L lead concentration resulting from an acidic water (pH = 4.2) dripping test and 4.1 mg/L concentration detected after a soak in water with a pH of 7.0. This difference is theorized by Chen et al. (2020) to be the result of the soluble lead leaching out of the module early in a rainstorm followed by dilution from the continued rain.^69^

The effects of acid rain on perovskite modules damaged by hail were simulated experimentally by Wan et al. (2021), using encapsulated 8 cm × 8 cm PSC minimodules.^70^ Three types of encapsulant were tested for their efficacy in preventing both physical damage and lead leachate. Modules were subjected to simulated hail using 15 mm or 25 mm diameter balls of ice. The 15 mm ice balls caused very little damage, whereas the 25 mm ice balls cracked or broke a significant portion of the minimodules. All modules were then soaked in acidic water (pH = 5.6) containing HCl and H_2_SO_4_. Analysis showed the lead content of the 5 cm of soil beneath the modules would increase only 3.4 ppm.^70^ This is considered an acceptable level based on guidance from the US EPA that indicates under 100 ppm is safe for growing root vegetables and under 400 ppm is safe for children to play in.^71^

Simulated rainwater was also used to test leaching of unencapsulated and exposed thin film perovskite layers in Hailegnaw et al. (2015),^7^ where an acidic solution containing HNO_3_ (pH = 4.2), a deionized water solution (pH = 6.0), and a basic solution containing NaOH (pH = 8.2) is used to determine the removal of lead from the films. For each of the artificial rainwater formulations, 67%–72% of the perovskite layer solubilized after one hour of continual exposure. This correlates to a lead concentration increase of approximately 70 ppm in the top 1 cm of soil, much higher than the value calculated for the top 5 cm of soil in Wan et al. (2020) that tested pristine encapsulated modules that were damaged by hail.^70^

### Fire

In the case of fires, PV modules are exposed to high temperatures, which can damage the modules and release Pb into the air or into the water used to extinguish the fire. In Conings et al. (2019) fire exposure experiments quantified the percentage of lead released from a perovskite PV module during a fire.^72^ Analysis of nearby surfaces was assessed to determine the amount of lead carried by the smoke and deposited on surfaces in the vicinity of the fire. The glass encapsulation mostly absorbed the Pb from the perovskite layer of the module, and the Pb found on nearby surfaces was released from exposed sections of PV modules where the encapsulation was damaged or destroyed.^72^ Polymer encapsulation layers could pose an elevated risk in the case of fire due to the lower temperatures required to incinerate polymeric materials compared with glass, resulting in exposure of the perovskite layer if the glass were to break or the edge seal were to fail.

A computational dispersion modeling study assessed rooftop fires of crystalline silicon modules to evaluate the risk of aerosolized lead from the lead-tin solder using the US EPA’s Guassian plume dispersion model SCREEN3.^73^ The percentage of lead released from the modules was set at 4.6%, a value taken from Prume et al. (2015).^74^ The study assessed a matrix of three fire sizes and five times to extinguish. Assuming worst-case scenarios for each, the level of lead in the simulated plume was computed and compared with the Acute Exposure Guidelines (AEGL) from the US EPA as well as the Protective Action Criteria (PAC) from the US DOE. For all combinations of fire sizes and fire times, the airborne lead content was below the AEGL and PAC limits.^73^ This study did not include perovskite PV; however, it is a starting point to consider the airborne lead risk from a fire. Additional fire testing is needed to collect module-specific emission data, which is currently lacking in published data.

### Leaching

Leaching is the primary route of contaminant migration from the internal layers of the PSC to the external environment. This can occur when the inside of the module (the perovskite and possibly lead-tin solder) is exposed to a liquid, such as rainwater, water from extinguishing fires, or floodwater. The contaminants can then dissolve into the water forming ions, which can leach out of the module through cracks or module edges and into groundwater, soil, or be absorbed by nearby plants. There are two general categories of leaching tests: batch and column tests. Batch tests involve mixing an extraction fluid with module pieces, followed by agitation for 12–48 hours. Column test methods are longer term, averaging one month in duration, where the module pieces are under continuous flow of leaching solution in a column. We focus on batch tests because they are used by regulatory bodies to determine applicability of regulations and to make decisions on how to handle waste.

The TCLP batch test involves subjecting a 100–110 gram sample that can entirely pass through a 9.5 mm standard sieve to 18 ± 2 h of end-over-end agitation in a 20:1 mass ratio of extraction fluid. Two extraction fluids are used in the TCLP, and details can be found in the full procedure documentation.^60^ Following the agitation period, the waste and extraction fluid mixture are then filtered through a 0.6–0.8 μm sieve to separate out solids and then analyzed for target compounds.^60^ The 20:1 ratio of extraction liquid to module pieces is based on mass, meaning the composition and density of the module is an important factor in the leaching test results. For example, use of a thick glass substrate will result in a smaller active area of the module needed to reach the 100 g sample size, versus a thinner glass or lightweight polymeric substrate, which will require a larger active area of the module to reach the same mass (i.e., more lead-containing perovskite material). If the leaching behavior is controlled by the available perovskite content, then leaching from a polymeric substrate would result in higher concentrations than a thick glass substrate. If leaching is controlled by the solubility limit of the perovskite layer, then the difference in module material densities would not result in differences in leachate concentrations.

A different approach to testing leaching potential is the synthetic precipitation leaching procedure (SPLP).^75^ The primary difference between TCLP and SPLP is that the SPLP protocol evaluates the release of chemicals and compounds during standard rainfall that then contaminate soil and groundwater. Both the TCLP and SPLP were designed to simulate 100 years in a landfill.^76^ ASTM has developed a test similar to the TCLP, called the water leaching test (WLT), titled “ ASTM D3987 Standard Test Method for Shake Extraction of Solid Waste with Water.”^77^ The WLT is not used by the EPA or any states to determine the applicability of regulations but is a useful comparison, nonetheless. In addition, California uses a Waste Extraction Test (WET) to determine applicability of state level regulations.^78^ The primary differences between these tests are the chemistry of the extraction fluid and liquid to solid mass ratio. Table 2 summarizes the differences in extraction fluids used in the four leaching tests presented here.

**Table 2:** Comparison of leaching tests' extraction fluid compositions

Test	Extraction fluid	pH	Liquid to solid mass ratio
TCLP	#1: Acetic acid, sodium hydroxide, distilled water	4.93	20:1
	#2: Acetic acid and distilled water	2.88	
SPLP	Sulfuric acid, nitric acid, distilled water	4.2	20:1
WLT	Deionized water	7.1	10:1
WET	Citric acid and sodium hydroxide, distilled water	4.0	10:1

Table 3 provides an overview of perovskite PV leaching data available in published peer-reviewed studies, including standardized tests such as the TCLP alongside non-standard tests such as the artificial rainwater testing.

**Table 3:** Collection of published leaching data for perovskite solar cells and modules

Test	Perovskite	pH	Contact with fluid	Broken	Encapsulated	Substrate	Sequestration	Lead (mg/L)	Ref	Notes
TCLP	MAPbI_3_	4.9	Soaked	No	No	Glass	No	4.2	Panthi et al.^79^	
TCLP	MAPbI_3_	4.9	Tumbled	No	Yes, 4mm	Glass	No	0.96	Panthi et al.^79^	
TCLP	MAPbI_3_	4.9	Tumbled	No	Yes, 10mm	Glass	No	0.3	Panthi et al.^79^	
TCLP	Unspecified MHP	4.9	Tumbled	Yes	No	Glass	No	3.4	Su et al.^80^	
TCLP	Unspecified MHP	4.9	Tumbled	Yes	No	Glass	No	4.6	Su et al.^80^	10:1 ratio
TCLP	Unspecified MHP	4.9	Tumbled	Yes	No	Glass	No	*6*.*1*	Su et al.^80^	5:1 ratio
SPLP	Unspecified MHP	3.2	Tumbled	Yes	No	Glass	No	*5*.*6*	Su et al.^80^	10:1 ratio
WLT	Unspecified MHP	7.1	Tumbled	Yes	No	Glass	No	3.0	Su et al.^80^	10:1 ratio
TCLP	(FA_0.92_MA_0.08_)_0.9_ Cs_0.1_Pb(I_0.92_Br_0.08_)_3_	4.9	Tumbled	Yes	Yes	Glass	No	*9*.*8*	Li et al.^81^	
TCLP	(FA_0.92_MA_0.08_)_0.9_ Cs_0.1_Pb(I_0.92_Br_0.08_)_3_	4.9	Tumbled	Yes	Yes	Polymer	No	*65*	Li et al.^81^	
TCLP	(FA_0.92_MA_0.08_)_0.9_ Cs_0.1_Pb(I_0.92_Br_0.08_)_3_	4.9	Tumbled	Yes	Yes	Glass	Yes	2.5	Li et al.^81^	
TCLP	(FA_0.92_MA_0.08_)_0.9_ Cs_0.1_Pb(I_0.92_Br_0.08_)_3_	4.9	Tumbled	Yes	Yes	Polymer	Yes	2.3	Li et al.^81^	
TCLP	(FA_0.92_MA_0.08_)_0.9_ Cs_0.1_Pb(I_0.92_Br_0.08_)_3_	4.9	Tumbled	No	No	Glass	No	*16*	Li et al.^81^	
TCLP	(FA_0.92_MA_0.08_)_0.9_ Cs_0.1_Pb(I_0.92_Br_0.08_)_3_	4.9	Tumbled	No	No	Polymer	No	*127*	Li et al.^81^	
TCLP	(FA_0.92_MA_0.08_)_0.9_ Cs_0.1_Pb(I_0.92_Br_0.08_)_3_	4.9	Tumbled	No	No	Glass	Yes	4.0	Li et al.^81^	
TCLP	(FA_0.92_MA_0.08_)_0.9_ Cs_0.1_Pb(I_0.92_Br_0.08_)_3_	4.9	Tumbled	No	No	Polymer	Yes	4.8	Li et al.^81^	
TCLP	FA_0.95_MA_0.05_ Pb(I_0.95_Br_0.05_)_3_	4.9	Tumbled	No	No	Glass	No	*14*.*2*	Moody et al.^82^	
TCLP	FA_0.95_MA_0.05_ Pb(I_0.95_Br_0.05_)_3_	4.9	Tumbled	No	No	Polymer	No	*713*	Moody et al.^82^	
TCLP	Unspecified MHP	4.9	Tumbled	Yes	No	Polymer	No	*412*	Li et al.^83^	
TCLP	Unspecified MHP	4.9	Tumbled	Yes	Yes	Polymer	No	*199*	Li et al.^83^	
TCLP	Unspecified MHP	4.9	Tumbled	Yes	No	Polymer	Yes	0.8	Li et al.^83^	
TCLP	Unspecified MHP	4.9	Tumbled	Yes	Yes	Polymer	Yes	0.6	Li et al.^83^	
TCLP	FA_0.95_MA_0.05_ Pb(I_0.95_Br_0.05_)_3_	4.9	Tumbled	Yes	Yes	Glass	No	*10*.*0*	Moody et al.^84^	
TCLP	MAPbI_3_	4.9	Tumbled	Yes	Yes	Glass	No	*21*.*9*	Moody et al.^84^	Thin glass
NS	(CsPbI_3_)_0.05_(MAPbBr_3_)_0.15_(FAPbI_3_)_0.8_	4.2	Dripped	Yes	Yes	Glass	No	*19*.*4*	Li et al.^85^	n-i-p, 5 mL/hr drip
NS	(CsPbI_3_)_0.05_(MAPbBr_3_)_0.15_(FAPbI_3_)_0.8_	4.2	Dripped	Yes	Yes	Glass	Yes	0.002	Li et al.^85^	n-i-p, 5 mL/hr drip
NS	(CsPbI_3_)_0.05_(MAPbBr_3_)_0.15_(FAPbI_3_)_0.8_	4.2	Dripped	Yes	Yes	Glass	No	*28*.*9*	Li et al.^85^	p-i-n, 5 mL/hr drip
NS	(CsPbI_3_)_0.05_(MAPbBr_3_)_0.15_(FAPbI_3_)_0.8_	4.2	Dripped	Yes	Yes	Glass	Yes	0.0025	Li et al.^85^	p-i-n, 5 mL/hr drip
NS	(CsPbI_3_)_0.05_(MAPbBr_3_)_0.15_(FAPbI_3_)_0.8_	7.0	Soaked	Yes	Yes	Glass	No	*12*.*9*	Li et al.^85^	n-i-p, 7 day soak
NS	(CsPbI_3_)_0.05_(MAPbBr_3_)_0.15_(FAPbI_3_)_0.8_	7.0	Soaked	Yes	Yes	Glass	Yes	0.0093	Li et al.^85^	n-i-p, 7 day soak
NS	(CsPbI_3_)_0.05_(MAPbBr_3_)_0.15_(FAPbI_3_)_0.8_	7.0	Soaked	Yes	Yes	Glass	No	*11*.*6*	Li et al.^85^	p-i-n, 7 day soak
NS	(CsPbI_3_)_0.05_(MAPbBr_3_)_0.15_(FAPbI_3_)_0.8_	7.0	Soaked	Yes	Yes	Glass	Yes	0.0097	Li et al.^85^	p-i-n, 7 day soak
NS	Cs_0.07_FA_0.93_PbI_3_	4.2	Dripped	Yes	No	Glass	No	*16*.*8*	Jiang et al.^86^	
NS	Cs_0.07_FA_0.93_PbI_3_	4.2	Dripped	Yes	Yes	Glass	No	*16*.*2*	Jiang et al.^86^	
NS	Cs_0.07_FA_0.93_PbI_3_	4.2	Dripped	Yes	Yes	Glass	Yes	2.4	Jiang et al.^86^	Surlyn polymer encapsulant
NS	Cs_0.07_FA_0.93_PbI_3_	4.2	Dripped	Yes	Yes	Glass	Yes	0.9	Jiang et al.^86^	Epoxy resin encapsulant
NS	Cs_0.07_FA_0.93_PbI_3_	4.2	Dripped	No	Yes	Glass	Yes	0.05	Jiang et al.^86^	
NS	(CsPbI_3_)_0.05_(MAPbBr_3_)_0.15_(FAPbI_3_)_0.85_		Soaked	Yes	Yes	Glass	No	*9*.*5*	Li et al.^87^	Front and back damage
NS	(CsPbI_3_)_0.05_(MAPbBr_3_)_0.15_(FAPbI_3_)_0.85_	4.2	Soaked	Yes	Yes	Glass	No	*7*.*1*	Li et al.^87^	Front damage
NS	(CsPbI_3_)_0.05_(MAPbBr_3_)_0.15_(FAPbI_3_)_0.85_	4.2	Soaked	Yes	Yes	Glass	No	*5*.*7*	et al.^87^	Back damage
NS	(CsPbI_3_)_0.05_(MAPbBr_3_)_0.15_(FAPbI_3_)_0.85_	4.2	Soaked	Yes	Yes	Glass	Yes	1.1	Li et al.^87^	Back coating, back damage
NS	(CsPbI_3_)_0.05_(MAPbBr_3_)_0.15_(FAPbI_3_)_0.85_	4.2	Soaked	Yes	Yes	Glass	Yes	0.3	Li et al.^87^	Front coating, front damage
NS	(CsPbI_3_)_0.05_(MAPbBr_3_)_0.15_(FAPbI_3_)_0.85_	4.2	Soaked	Yes	Yes	Glass	Yes	0.3	Li et al.^87^	Both sides coating, both sides damaged
NS	Cs_0.05_(FA_0.9_MA_0.1_)_0.95_Pb(I_0.9_Br_0.1_)	7.0	Soaked	No	No	Glass	No	2.9	eNiu t al.^88^	
NS	Cs_0.05_(FA_0.9_MA_0.1_)_0.95_Pb(I_0.9_Br_0.1_)	7.0	Soaked	No	No	Glass	Yes	1.33	Niu et al.^88^	20 mg/mL AAm
NS	Cs_0.05_(FA_0.9_MA_0.1_)_0.95_Pb(I_0.9_Br_0.1_)	7.0	Soaked	No	No	Glass	Yes	0.74	Niu et al.^88^	80 mg/mL AAm
NS	Rb_0.05_Cs_0.05_FA_0.85_MA_0.05_PbI_2.85_Br_0.15_	7.0	Dripped	Yes	Yes	Glass	No	*13*.*24*	Chen et al.^69^	
NS	Rb_0.05_Cs_0.05_FA_0.85_MA_0.05_PbI_2.85_Br_0.15_	4.2	Dripped	Yes	Yes	Glass	No	*15*.*5*	Chen et al.^69^	
NS	Rb_0.05_Cs_0.05_FA_0.85_MA_0.05_PbI_2.85_Br_0.15_	7.0	Dripped	Yes	Yes	Glass	Yes	1.92	Chen et al.^69^	
NS	Rb_0.05_Cs_0.05_FA_0.85_MA_0.05_PbI_2.85_Br_0.15_	4.2	Dripped	Yes	Yes	Glass	Yes	2.55	Chen et al.^69^	
NS	Rb_0.05_Cs_0.05_FA_0.85_MA_0.05_PbI_2.85_Br_0.15_	6	Dripped	Yes	Yes	Glass	No	0.432	Chen et al.^69^	Outdoor rain test
NS	Rb_0.05_Cs_0.05_FA_0.85_MA_0.05_PbI_2.85_Br_0.15_	6	Dripped	Yes	Yes	Glass	Yes	0.033	Chen et al.^69^	Outdoor rain test
NS	Rb_0.05_Cs_0.05_FA_0.85_MA_0.05_PbI_2.85_Br_0.15_	7.0	Soaked	Yes	Yes	Glass	No	4.1	Chen et al.^69^	
NS	Rb_0.05_Cs_0.05_FA_0.85_MA_0.05_PbI_2.85_Br_0.15_	7.0	Soaked	Yes	Yes	Glass	Yes	0.45	Chen et al.^69^	
NS	MAPbI_3_	4.2	Dripped	No	No	Glass	No	*11*.*8*	Hailegnaw et al.^7^	
NS	MAPbI_3_	6.0	Dripped	No	No	Glass	No	*12*.*2*	Hailegnaw et al.^7^	
NS	MAPbI_3_	8.1	Dripped	No	No	Glass	No	*13*.*2*	Hailegnaw et al.^7^	

The test designation NS represents non-standardized test protocols. Lead concentration values exceeding the 5 mg/L threshold are in red italics.

It can be useful to compare the leaching data from PSCs with commercially available silicon PV modules. Panthi et al. (2021) performed leaching tests on small PSCs and crystalline silicon PV modules using the TCLP procedure, showing that Pb was released from undamaged encapsulated PSCs at a level of approximately 0.3–1 mg/L in acidic conditions. This value remained constant for all combinations of broken or unbroken unencapsulated cells in agitated or still leaching solution.^79^ Encapsulation was demonstrated to be very influential, as the unencapsulated module samples leached around 4 mg/L of lead in the TCLP test. The importance of encapsulation is echoed in other publications as well.^79^^,^^81^^,^^83^ In comparison, the two commercially available Si modules leached up to 9.1 mg/L of lead due to the use of lead solder, but details of the sampling method were not documented and results should not be directly compared with the prototype PSCs.^79^

TCLP test data have shown inconsistency with the levels of lead leached from both unencapsulated and damaged encapsulated modules with glass substrates.^7^^,^^69^^,^^79^^,^^81^^,^^83^^,^^84^^,^^85^^,^^86^^,^^87^^,^^88^ Unencapsulated modules have been reported to leach lead in the range of 2.9 mg/L, published in Niu et al. (2021),^88^ to 16.8 mg/L, published in Jiang et al. (2019).^86^ Broken encapsulated modules have a similar range, from 3.4 mg/L in Su et al. (2020)^80^ to 28.9 mg/L in Li et al. (2021).^85^ It should be noted that more data points are available for the broken encapsulated modules than the unencapsulated modules.

Su et al. (2020) compared the results of the TCLP, SPLP, and WLT on unencapsulated PSCs, showing that the SPLP test resulted in the highest measurements of Pb leaching, a value of 5.5 mg/L.^80^ The WLT measured a leached concentration of 3.0 mg/L. The TCLP test varied considerably based on the solid to liquid ratio. Su et al. (2020) tested mass ratios of 20:1, 10:1, and 5:1, showing the leaching values increase as the ratio decreases. The 5:1 ratio measured a Pb concentration of 6.1 mg/L, whereas the standard 20:1 ratio measured 3.2 mg/L^80^

TCLP tests were also performed by Li et al. (2022),^81^ Moody et al. (2020),^82^ and Li et al. (2021).^83^ Li et al. (2022) showed that the extraction fluid from tests of broken unencapsulated modules on glass substrates contains 16 mg/L of lead and those from tests using polymer substrates contain 127 mg/L of lead.^81^ Moody et al. (2020) reported similar results of 14.2 mg/L and 713 mg/L of lead in the TCLP leaching fluid for unencapsulated modules on glass and polymer substrates, respectively.^82^ Li et al. (2021) showed that unencapsulated polymer modules released 412 mg/L of lead in the TCLP test, and the addition of encapsulation reduced the release by about half, to 199 mg/L.^83^ These data highlight the importance of substrate weight in the TCLP test data.

Leach testing provides insight into the potential for lead to contaminate soil and groundwater and potentially harm surrounding communities and ecosystems. The variety of different testing methods used to evaluate risk from leaching indicates a wide range in the data, adding uncertainty to the lead safety of PSCs. Given the harm that results from lead contamination, the risk must be more fully understood before PSC module commercialization.

However, leaching studies help to define only part of the risk from lead releases. As discussed previously, the form and valence state of the lead ions and compounds in solution plays an important role in its risk profile. Pb^2+^ appears to be significantly more bioavailable and could enter the food chain more easily. More research is needed on which valance states of lead released from perovskites are warranted.

## Approaches to reducing releases

Several publications have demonstrated the concept of adding a lead sequestering polymer layer inside the module to trap lead before it can leach out. Chen et al. (2020) achieved a 62-fold reduction in lead leaching by adding a cation-exchange resin layered on the glass surface of the module.^69^ Li et al. (2020) applied a film containing phosphonic acids to the front glass surface as well as added a polymer film blended with lead-chelating agents to the metal electrode side, resulting in a 25-fold reduction of leached lead after the PSCs were soaked in an artificial rainwater solution (pH = 4.2) at both room temperature and 50°C.^87^ This approach to sequestration and entrapment of the lead is a useful solution to ensure that lead does not easily escape the module.

Jiang et al. (2019) encapsulated a PSC module with an epoxy resin that exhibits self-healing characteristics around 42°C.^86^ Modules were subjected to a series of 45 mm metal balls dropped onto the modules to simulate hail. Data showed that the epoxy layer significantly reduced the Pb releases during leaching tests. The use of this self-healing epoxy could be very helpful in the instance of module damage after severe weather, such that the cracks in the module could heal themselves, reducing worker exposure when the module is removed.

**Figure 4:**
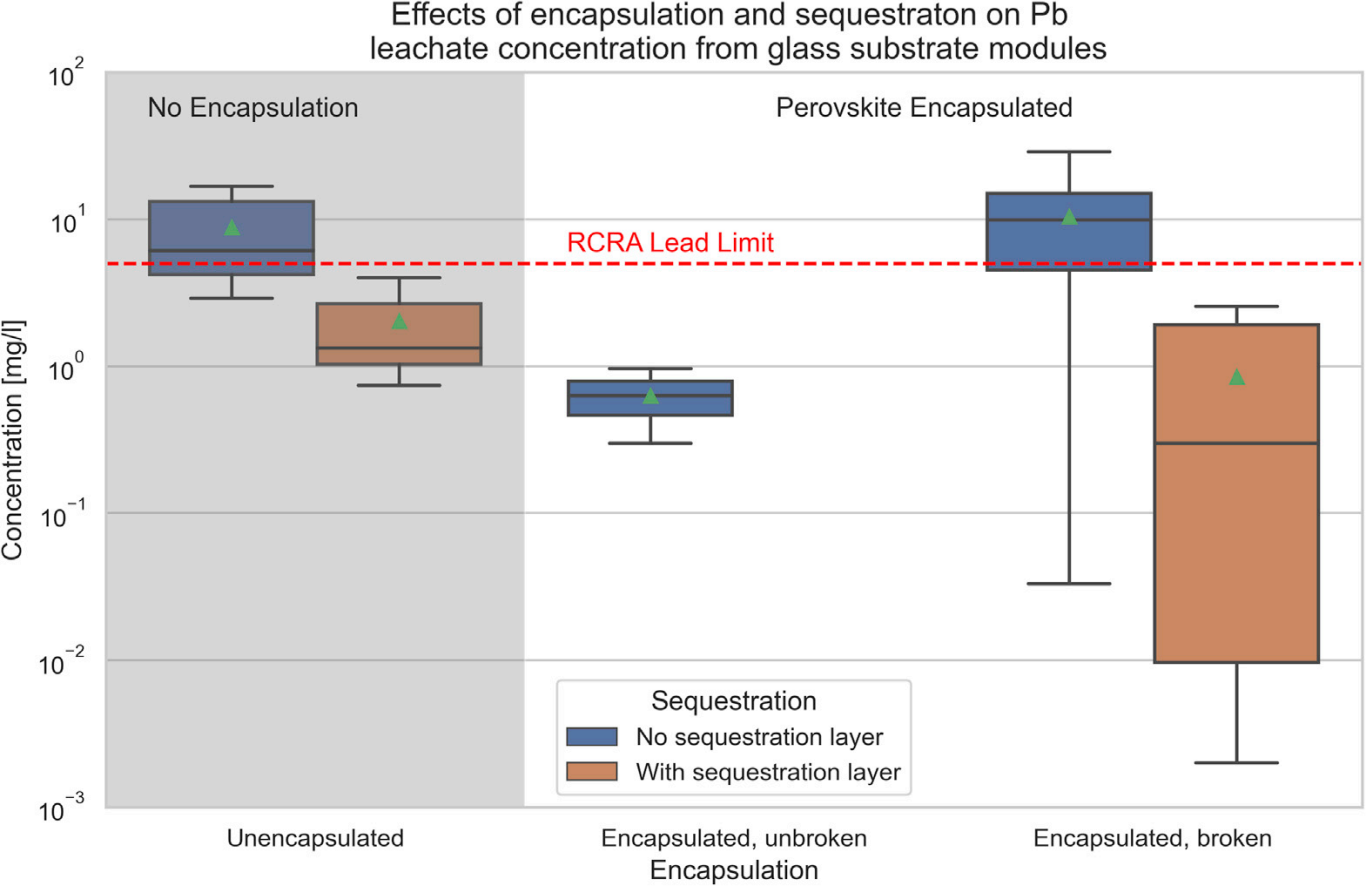
Leaching data of glass substrate perovskite cells and modules with and without encapsulation Unencapsulated and broken encapsulated modules shown with and without sequestration layers. Data from Table 3. Boxes show the range from the 25th to 75th percentiles. Triangles and horizontal lines indicate the mean and median, respectively. Whiskers show the range of the data.

**Figure 5:**
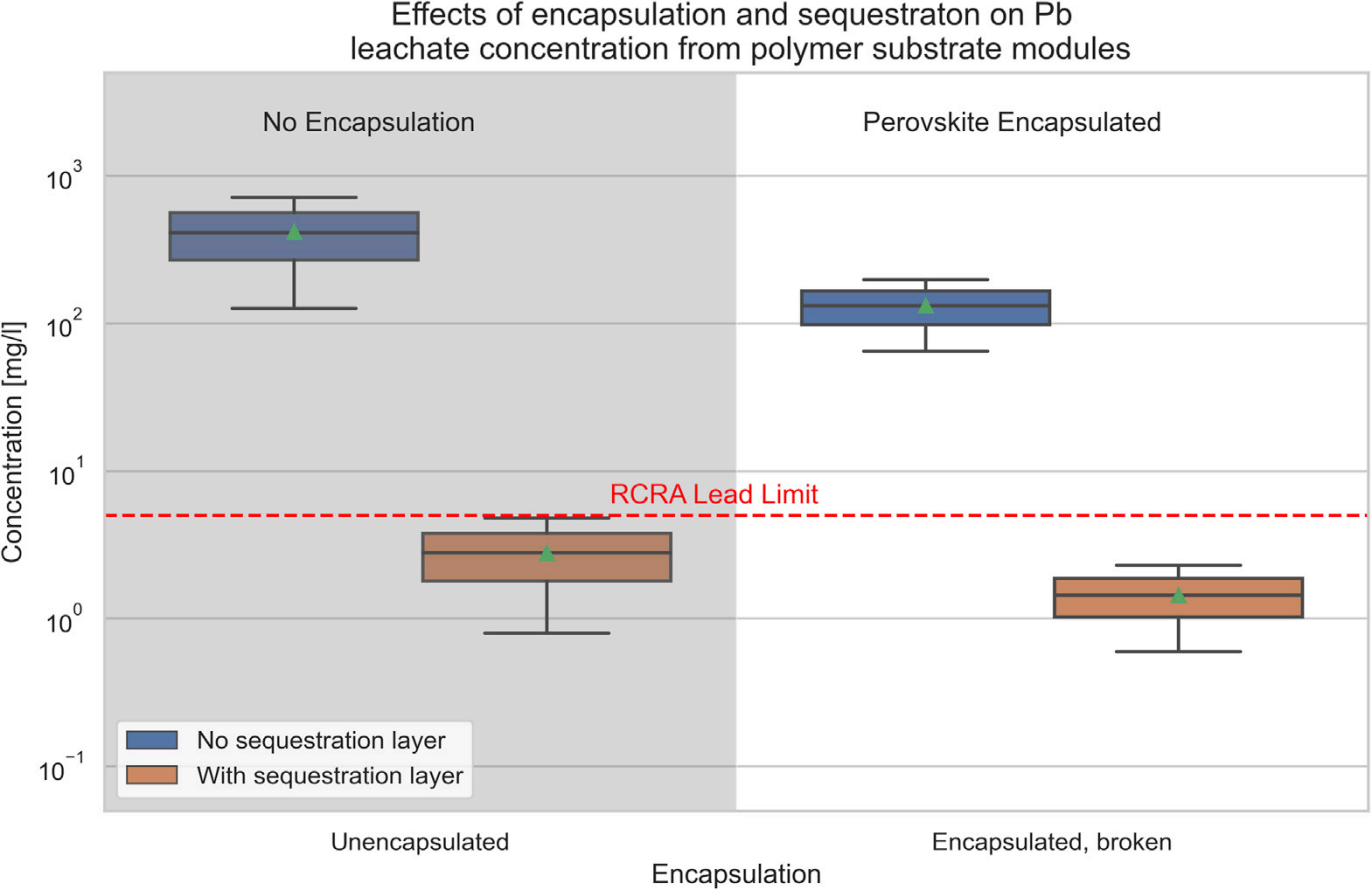
Leaching data of polymer substrate perovskite cells and modules with and without encapsulation and sequestration layers Data from Table 3. Boxes show the range from the 25th to 75th percentiles. Triangles and horizontal lines indicate the mean and median, respectively. Whiskers show the range of the data.

Figure 4 summarizes the published leaching data on glass substrate PSCs. Two key takeaways can be observed. The first is that encapsulation does not mitigate lead leaching. If water can get under the encapsulation layer, then lead leaching occurs at levels equivalent to unencapsulated cells. Second, sequestration technology is effective. Several sequestration methods have been published, each proving to be highly effective at reducing lead release from modules.^83^^,^^85^^,^^86^^,^^87^^,^^88^ This is also seen in the data for polymer substrate PSCs, shown in Figure 5, where the cells with sequestration layers average 2.8 mg/L and 1.45 mg/L for unencapsulated and damaged encapsulated cells, respectively. Comparably, the polymer substrate cells without sequestration average 417 mg/L and 132 mg/L for unencapsulated and damaged encapsulated cells, respectively.

### Lead-free perovskites

**Figure 6:**
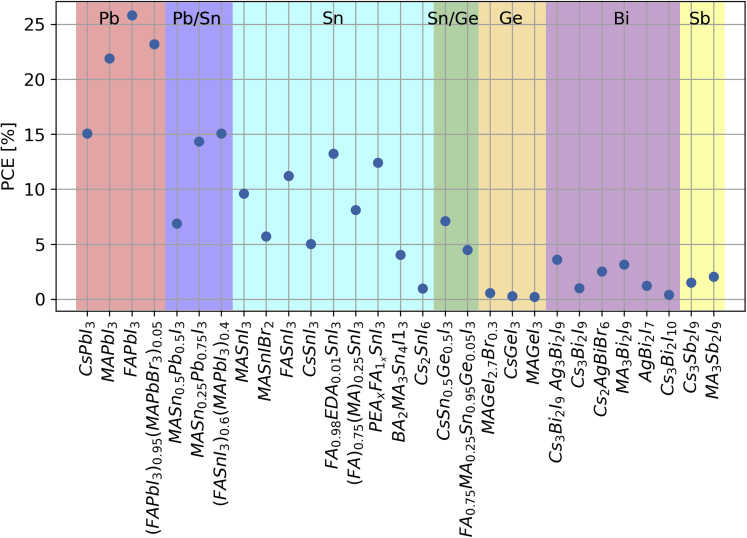
Perovskite PSC compositions and their highest reported associated efficiencies Data are grouped by the metal present in the metal halide perovskite formula^1^^,^^4^^,^^90^^,^^91^^,^^92^^,^^93^^,^^94^^,^^95^^,^^96^^,^^97^^,^^98^^,^^99^^,^^100^^,^^101^^,^^102^^,^^103^^,^^104^^,^^105^^,^^106^^,^^107^^,^^108^^,^^109^ (Table S1 lists the data and sources for all points).

A current area of research is focused on removing or reducing the amount of Pb in PSCs. A “low-lead” alloyed approach is being investigated, using perovskite formulas containing both tin and lead in the form AB^I^_y_B^II^_1-y_X_3_. The tin-lead PSC compositions show efficiencies that are approaching those of lead PSCs. These formulations show potential to overcome some of the hurdles faced by tin PSCs and reduce the amount of lead; however, instability and short lifetimes remain a serious problem.^89^ Figure 6 shows a variety of PSC compositions, grouped by metal, and the maximum measured efficiency of each.

Although low-lead and lead-free perovskites offer a route to avoid lead release, they have several important shortcomings. Ge PSCs have not surpassed 0.6% PCE and have short stability lifetimes.^99^ Tin perovskites have a higher theoretical efficiency similar to lead perovskites; however, the Sn-based cells have consistently shown both lower efficiencies and shorter air stability times.^109^^,^^110^^,^^111^ The best performing tin PSCs were tested in nitrogen environments because tin oxidizes quickly in the presence of oxygen, causing degradation of the perovskite; however, recent progress to increase the stability of tin perovskites has been reported.^112^ Lead has thus far offered the best combination of efficiency and stability. Therefore, the most likely path to commercialization will use lead halide perovskites and technologies that sequester and immobilize the lead. This path offers the greatest potential for reducing leaching risks.

### Recycling

**Figure 7:**
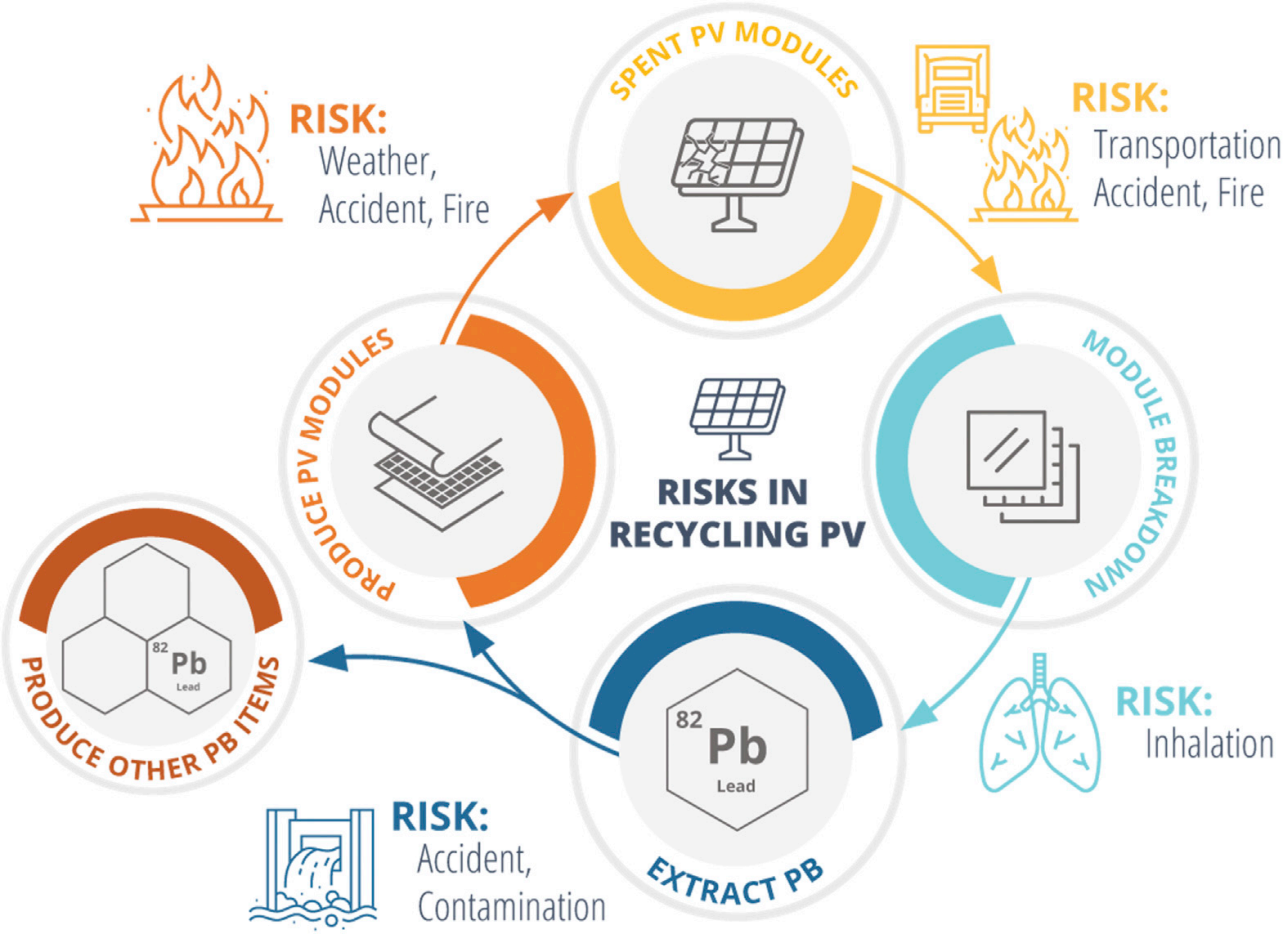
Circular recycling of perovskite PV modules to divert panels from landfills and reuse the lead, glass, and other materials in the production of new modules or other products, such as lead acid batteries Risks at each stage are identified.

The development of a robust PSC recycling program would reduce the environmental impact of PSC modules by proactively preventing landfill disposal. One approach is to enact legislation that requires PV retailers to takeback and recycle used modules, as was recently done by the state of Washington.^64^ It is likely that more states will follow suit in the future. Figure 7 shows a circular process in which recycling of spent modules keeps lead and other materials out of the environment by diverting modules away from landfills and reusing the materials to make new modules or other products such as batteries. Although this recycling process mitigates the leaching risk of modules in landfills, it is not without risk itself. In Figure 7, the risks at each step are identified. The transportation of spent modules involves the risk of accident or fire. This is not an additional risk associated with recycling, as it is also present in the transportation of new modules to customers. In the module breakdown and material recovery process, shredding, crushing, or grinding of the modules could release lead dust but these risks are manageable with proper safety equipment and protocols. Overall, recycling modules reduces the risk of lead leaching into the soil and groundwater through landfills; however, the inherent risks of recycling and other downstream processes need to be carefully managed.

Recycling of PSC module components and reuse of recovered materials in the fabrication of new PSC modules is an active area of study. Recovery of substrate materials at lab scale and subsequent reuse in fabrication of PSCs has been successfully demonstrated for a variety of PSC compositions and architectures without compromising performance.^113^ Perovskite layers and lead can be removed through dissolution using N-Dimethylformamide (DMF), chlorobenzene, KOH, or a eutectic solvent. Chen et al. explored recycling processes for lead iodide perovskite formulations and suggested that reusable adsorbents may be important for recycling to be cost effective.^114^ A previous study had investigated use of iron-incorporated hydroxyapatite to separate and adsorb lead, but the composite dissolves during lead release, preventing reuse.^115^ Chen et al. instead demonstrated a thermal process to delaminate intact modules. Then, after the glass substrates were separated, an organic solvent was used to dissolve the perovskite layer, and a carboxylic acid cation-exchange resin adsorbed lead as Pb(NO_3_)_2_. The lead was then precipitated as PbI_2_.^114^ The weakly acidic cation resin (WAC) was determined to be more efficient at liberating lead than a strongly acidic cation-exchange resin.^69^^,^^116^ Three 1-h treatments in a WAC-gel that can be regenerated resulted in conversion of 99.6% of the Pb(NO_3_)_2_ to PbI_2_. This method potentially enables reuse of both the glass substrate and PbI_2_. PbI_2_ recovered from perovskite modules and used in the fabrication of new perovskite modules was shown to result in comparable efficiency to modules fabricated with commercial high-purity PbI_2_. Recycling, material recovery, and reuse need to be demonstrated and optimized for large-area substrates and other compositions and architectures for commercialization and scale-up.^113^ Depending on the purity of recovered material, open-loop recycling, in which materials are used to create other types of products, may be appropriate. The environmental impacts of the recycling processes themselves, which require energy and chemical usage and produce emissions, also require further study.

Cost also plays a significant role in the decision to recycle. The National Renewable Energy Laboratory (NREL) estimates the cost of fully recycling a c-Si module at about $20–30 per module, whereas disposal in a standard landfill is about 10% of the cost at $0.5–2 per module.^117^ Disposal of a module in a hazardous waste facility is higher at around $3–4; smaller quantities of modules with high lead content can be charged over 100x more.^118^ Moreover, these values do not include shipping costs. Most recycling of c-Si modules is performed on existing glass and metal recycling lines that are not customized for PV and do not recover high-value materials such as silver and silicon.^119^ Modules are generally processed in batches and achieve roughly 78% material recovery by weight. In the EU, recycling facilities customized for c-Si became operational starting in 2019 and report recovery rates over 90%, including recovery of high-value silicon and silver.^47^ First Solar has offered commercial-scale recycling for its thin-film CdTe modules for over 10 years (∼4,500 tons were processed in 2017) to prevent the Cd and Te in its modules from ending up in a landfill.^47^ The company reports 90%–95% material recovery.^120^ First Solar is the first and only US company to offer take-back services and recycling, but this approach is not standard among currently operating PV module manufacturers. Widespread adoption of solar modules began in the early 2000s, so most deployed systems are still operating, meaning that disposal and recycling demand is only going to grow. Recycle PV Solar, a PV recycling company, estimates that, currently, around 10% of solar modules are recycled.^121^

Life cycle assessment (LCA) can be used to compare landfill and recycling end-of-life scenarios. Tian et al. assessed cradle-to-grave and cradle-to-cradle environmental impacts for six solvent-processed PSC module architectures. Energy payback time (EPBT) estimates were 0.09 to 0.93 years for the recycling scenarios, compared with 0.19 to 1.1 for modules that are landfilled.^122^ Use of precious metals, such as gold and silver, was found to have severe environmental effects and was considered unsustainable for high-volume manufacturing. Recycling and lifetime extension, through enhanced device stability, were identified as key factors for improving sustainability.

## Conclusions and recommendations

In this work, we review the environmental and health risks of lead halide PSCs. From this, we identify several key research gaps, highlighting the information needed to safely commercialize this technology.

The first gap is the lack of consistency between the data collected from leaching tests. Some testing protocols have indicated a high level of lead in leachate, whereas others do not. In addition, the TCLP test result is impacted by sampling method, including sampling locations within the module; the composition, thickness, and density of the substrate; cutting method; and amount of crushing or other variations in the sample preparation. Future work should carefully specify the methods used to sample and prepare module material for testing. In addition, there are no data on the dermal absorption of perovskite dust, leaving unanswered questions about the risk posed to workers handling damaged modules. Finally, test data are needed to assess the potential for damaged modules in the field to cause soil or groundwater contamination.

Another important gap in the available data is fire safety of PSC modules, including data on full encapsulated modules and Gaussian plume modeling to estimate the risk of airborne contaminant downstream exposure. The potential for exposure to perovskite and lead-containing dust needs to be studied to understand how firefighters and workers handling fire-damaged modules can work safely.

Lead sequestration materials incorporated into the module construction offer a promising means to capture and immobilize lead that may otherwise escape a module. More research is needed to validate this technology, including understanding the stability of the polymer materials (e.g., many polymers degrade under UV radiation) and any impacts on PSC performance and reliability. Testing is needed to determine if the lead sequestering layers will last for the life of the module, which could be as long as 20–30+ years.

The pursuit of lead-free or “low-lead” PSCs as a safer alternative would also help to mitigate the risk of lead release, as they may offer a more environmentally safe route to high efficiency PSCs. However, current challenges in this area include raising the efficiency and increasing stability of these alternative designs. Given the fundamental challenges of lead-free perovskite formulations, this may not be the most expedient route to mitigating lead risks of PSCs.

Finally, longer module lifetimes and recycling programs will be key components of reducing environmental impacts by postponing end-of-life management and diverting damaged and spent modules away from landfills. This eliminates the risk of landfill leaching and captures the lead and other materials for reuse in new modules or other products.

## Limitations of the study

Because perovskite PV modules have yet to be commercialized, we have had to assume that they will be produced at scale using designs and form factors typical of existing solar modules (glass-glass or glass-polymer laminates). The final designs and materials used for such modules may result in modifications to their associated risk profiles, as discussed in this review paper.

## References

[1] Llanos M., Yekani R., Demopoulos G.P., Basu N. (2020). Alternatives assessment of perovskite solar cell materials and their methods of fabrication. Renew. Sustain. Energy Rev..

[2] Jamal M., Bashar M., Hasan A.M., Almutairi Z.A., Alharbi H.F., Alharthi N.H., Karim M.R., Misran H., Amin N., Sopian K.B., Akhtaruzzaman M. (2018). Fabrication techniques and morphological analysis of perovskite absorber layer for high-efficiency perovskite solar cell: a review. Renew. Sustain. Energy Rev..

[3] Kojima A., Teshima K., Shirai Y., Miyasaka T. (2009). Organometal halide perovskites as visible-light sensitizers for photovoltaic cells. J. Am. Chem. Soc..

[4] Min H., Lee D.Y., Kim J., Kim G., Lee K.S., Kim J., Paik M.J., Kim Y.K., Kim K.S., Kim M.G. (2021). Perovskite solar cells with atomically coherent interlayers on SnO2 electrodes. Nature.

[5] National Renewable Energy Laboratory (2021).

[6] Sofia S.E., Wang H., Bruno A., Cruz-Campa J.L., Buonassisi T., Peters I.M. (2020). Roadmap for cost-effective, commercially-viable perovskite silicon tandems for the current and future PV market. Sustain. Energy Fuels.

[7] Hailegnaw B., Kirmayer S., Edri E., Hodes G., Cahen D. (2015). Rain on methylammonium lead iodide based perovskites: possible environmental effects of perovskite solar cells. J. Phys. Chem. Lett..

[8] Ravi V.K., Mondal B., Nawale V.V., Nag A. (2020). Don’t let the lead out: new material chemistry approaches for sustainable lead halide perovskite solar cells. ACS Omega.

[9] Chun-Ren Ke J., Walton A.S., Lewis D.J., Tedstone A., O'Brien P., Thomas A.G., Flavell W.R. (2017). In situ investigation of degradation at organometal halide perovskite surfaces by X-ray photoelectron spectroscopy at realistic water vapour pressure. Chem. Commun..

[10] Li Y., Xu X., Wang C., Wang C., Xie F., Yang J., Gao Y. (2015). Degradation by exposure of coevaporated CH3NH3PbI3 thin films. J. Phys. Chem. C.

[11] Zheng C., Rubel O. (2019). Unraveling the water degradation mechanism of CH3NH3PbI3. J. Phys. Chem. C.

[12] Angelidis T.N., Kydros K.A. (1995). Selective gold dissolution from a roasted auriferous pyrite-arsenopyrite concentrate. Hydrometallurgy.

[13] Clever H.L., Johnston F.J. (1980). The solubility of some sparingly soluble lead salts: an evaluation of the solubility in water and aqueous electrolyte solution. J. Phys. Chem. Ref. Data.

[14] Lichty D.M. (1903). The solubility of the chloride, the bromide, and the iodide of lead, in water, at temperatures from 0° upward. J. Am. Chem. Soc..

[15] National Center for Biotechnology Information. PubChem Compound Summary for CID 14793, Lead Dioxide; 2022.

[16] National Center for Biotechnology Information (2022).

[17] Babayigit A., Ethirajan A., Muller M., Conings B. (2016). Toxicity of organometal halide perovskite solar cells. Nat. Mater..

[18] Centers for Disease Control and Prevention (2021).

[19] National Institute for Occupational Safety and Health (NIOSH) (2021).

[20] Fewtrell L.K., Rachel, Pruss-Ustun A. (2003).

[21] Karri S.K., Saper R.B., Kales S.N. (2008). Lead encephalopathy due to traditional medicines. Curr. Drug Saf..

[22] Pan American Health Organization. Lead Contamination. www.paho.org/en/topics/lead; 2022.

[23] Canfield R.L., Henderson C.R., Cory-Slechta D.A., Cox C., Jusko T.A., Lanphear B.P. (2003). Intellectual impairment in children with blood lead concentrations below 10 microg per deciliter. N. Engl. J. Med..

[24] World Health Organization Lead Poisoning. http://www.who.int/news-room/fact-sheets/detail/lead-poisoning-and-health;2022.

[25] Benmessaoud I.R., Mahul-Mellier A.-L., Horváth E., Maco B., Spina M., Lashuel H.A., Forró L. (2016). Health hazards of methylammonium lead iodide based perovskites: cytotoxicity studies. Toxicol. Res..

[26] Patsiou D., del Rio-Cubilledo C., Catarino A.I., Summers S., Mohd Fahmi A., Boyle D., Fernandes T.F., Henry T.B. (2020). Exposure to Pb-halide perovskite nanoparticles can deliver bioavailable Pb but does not alter endogenous gut microbiota in zebrafish. Sci. Total Environ..

[27] World Health Organization (2021).

[28] Centers for Disease Control and Prevention Agency for Toxic Substances and Disease Registry Division of Toxicology (2005).

[29] Cooney J.J., Wuertz S. (1989). Toxic effects of tin compounds on microorganisms. J. Ind. Microbiol..

[30] Lanzetta L., Webb T., Zibouche N., Liang X., Ding D., Min G., Westbrook R.J.E., Gaggio B., Macdonald T.J., Islam M.S., Haque S.A. (2021). Degradation mechanism of hybrid tin-based perovskite solar cells and the critical role of tin (IV) iodide. Nat. Commun..

[31] Leijtens T., Prasanna R., Gold-Parker A., Toney M.F., McGehee M.D. (2017). Mechanism of tin oxidation and stabilization by lead substitution in tin halide perovskites. ACS Energy Lett..

[32] National Center for Biotechnology Information (2021). N.C.f.B. Information.

[33] National Center for Biotechnology Information PubChem Compound Summary for CID 62750, Cesium Hydroxide. pubchem.ncbi.nlm.nih.gov/compound/Cesium-hydroxide;2022.

[34] New Jersey Department of Health (2007). Hazardous Substance Fact Sheet Cesium Hydroxide..

[35] Agency for Toxic Substances and Disease Registry (ATSDR) (2013). What Are the Physiologic Effects of Chromium Exposure? Center for Disease Control. https://www.atsdr.cdc.gov/csem/chromium/physiologic_effects_of_chromium_exposure.html.

[36] Galvao J., Davis B., Tilley M., Normando E., Duchen M.R., Cordeiro M.F. (2014). Unexpected low-dose toxicity of the universal solvent DMSO. Faseb. J..

[37] Lee K.P., Chromey N.C., Culik R., Barnes J.R., Schneider P.W. (1987). Toxicity of N-methyl-2-pyrrolidone (NMP): teratogenic, subchronic, and two-year inhalation studies. Fund. Appl. Toxicol..

[38] Scailteur V., Lauwerys R.R. (1987). Dimethylformamide (DMF) hepatotoxicity. Toxicology.

[39] Foxall K. (2007).

[40] Zhang M., Xin D., Zheng X., Chen Q., Zhang W.-H. (2020). Toward greener solution processing of perovskite solar cells. ACS Sustainable Chem. Eng..

[41] Gardner K.L., Tait J.G., Merckx T., Qiu W., Paetzold U.W., Kootstra L., Jaysankar M., Gehlhaar R., Cheyns D., Heremans P., Poortmans J. (2016). Nonhazardous solvent systems for processing perovskite photovoltaics. Adv. Energy Mater..

[42] Schep L.J., Knudsen K., Slaughter R.J., Vale J.A., Mégarbane B. (2012). The clinical toxicology of gamma-hydroxybutyrate, gamma-butyrolactone and 1, 4-butanediol. Clin. Toxicol..

[43] Sinha P., Balas R., Krueger L., Wade A. (2012). Fate and transport evaluation of potential leaching risks from cadmium telluride photovoltaics. Environ. Toxicol. Chem..

[44] Fu Z., Xu M., Sheng Y., Yan Z., Meng J., Tong C., Li D., Wan Z., Ming Y., Mei A. (2019). Encapsulation of printable mesoscopic perovskite solar cells enables high temperature and long-term outdoor stability. Adv. Funct. Mater..

[45] Ma S., Yuan G., Zhang Y., Yang N., Li Y., Chen Q. (2022). Development of encapsulation strategies towards the commercialization of perovskite solar cells. Energy Environ. Sci..

[46] Uddin A., Upama M., Yi H., Duan L. (2019). Encapsulation of organic and perovskite solar cells: a review. Coatings.

[47] Heath G.A., Silverman T.J., Kempe M., Deceglie M., Ravikumar D., Remo T., Cui H., Sinha P., Libby C., Shaw S. (2020). Research and development priorities for silicon photovoltaic module recycling to support a circular economy. Nat. Energy.

[48] Kadro J.M., Hagfeldt A. (2017). The end-of-life of perovskite PV. Joule.

[49] Occupational Safety and Health Administration. Solvents. www.osha.gov/solvents; 2022.

[50] Occupational Safety and Health Administration (2014).

[51] Li J., Cao H.-L., Jiao W.-B., Wang Q., Wei M., Cantone I., Lü J., Abate A. (2020). Biological impact of lead from halide perovskites reveals the risk of introducing a safe threshold. Nat. Commun..

[52] Natasha S., Shahid M., Farooq A.B.U., Rabbani F., Khalid S., Dumat C. (2020). Risk assessment and biophysiochemical responses of spinach to foliar application of lead oxide nanoparticles: a multivariate analysis. Chemosphere.

[53] Filon F.L., Boeniger M., Maina G., Adami G., Spinelli P., Damian A. (2006). Skin absorption of inorganic lead (PbO) and the effect of skin cleansers. J. Occup. Environ. Med..

[54] U.S. Dept of Labor Occupational Safety and Health Administration. OSHA At-A-Glance. www.osha.gov/sites/default/files/publications/3439at-a-glance.pdf;2014.

[55] U.S. Dept. of Labor Occupational Safety and Health Administration. 1910.1153 - Silica Crystalline. www.osha.gov/laws-regs/regulations/standardnumber/1926/1926.1153; 2019.

[56] U.S. Dept. of Labor Occupational Safety and Health Administration. OSHA Technical Manual (OTM) Section III: Chapter 4 Heat Stress. www.osha.gov/otm/section-3-health-hazards/chapter-4; 2017.

[57] U.S. Dept. of Labor Occupational Safety and Health Adminstration.1910.1025 - Lead. www.osha.gov/laws-regs/regulations/standardnumber/1910/ 1910.1025; 2020.

[58] U.S. Environmental Protection Agency. National ambient air quality standards (NAAQS) for lead (Pb); 2016

[59] United States Environmental Protection Agency. Resource conservation and recovery act (RCRA). 42 U.S.C. §6901 et seq. www.govinfo.gov/content/pkg/USCODE-2011-title42/html/USCODE-2011-title42-chap82.htm; 1976.

[60] U.S. Environmental Protection Agency. Method 1311 Toxicity Characteristic Leaching Procedure. www.epa.gov/sites/default/files/2015-12/documents/1311.pdf; 1992.

[61] Federal Motor Carrier Safety Administration. Hazardous Waste 49 CFR 171.3. www.ecfr.gov/current/title-49/subtitle-B/chapter-I/subchapter-C/part-171/subpart-A/section-171.3; 2005.

[62] U.S. Comprehensive Environmental Response, Compensation, and Liability Act (CERCLA). www.govinfo.gov/content/pkg/USCODE-2011-title42/html/USCODE-2011-title42-chap103.htm; 1980. 10132830

[63] U.S. Environmental Protection Agency (2022). Lead at Superfund Sites. https://www.epa.gov/superfund/lead-superfund-sites.

[64] Chapter 70.355 RCW (2017). Washington Legislature.

[65] State of California (2021). Photovoltaic Modules (PV Modules) – Universal Waste Management Regulations. https://dtsc.ca.gov/photovoltaic-modules-pv-modules-universal-waste-management-regulations/.

[66] North Carolina Dept. of. Environmental Quality Environmental Management Commission. Final Report on the Activities Conducted to Establish a Regulatory Program for the Management and Decommissioning of. Renewable Energy Equipment; 2021.

[67] Reynolds W., Karmis M. (2019). Assessment of the Risks Associated with Thin Film Solar Panel Technology. The Virginia Center for Coal and Energy Research.. Virgnia Tech.

[68] Electric Power Research Institute (2021).

[69] Chen S., Deng Y., Gu H., Xu S., Wang S., Yu Z., Blum V., Huang J. (2020). Trapping lead in perovskite solar modules with abundant and low-cost cation-exchange resins. Nat. Energy.

[70] Wan J., Yu X., Zou J., Li K., Chen L., Peng Y., Cheng Y.-b. (2021). Lead contamination analysis of perovskite modules under simulated working conditions. Sol. Energy.

[71] U.S. Environmental Protection Agency (2020). Lead in Soil. http://www.epa.gov/lead.

[72] Conings B., Babayigit A., Boyen H.-G. (2019). Fire safety of lead halide perovskite photovoltaics. ACS Energy Lett..

[73] Sinha P., Heath G., Wade A., Komoto K. Human Health Risk Assessment Methods for PV, Part 1: Fire Risks (No. NREL/TP-6A20-72141). National Renewable Energy Lab.(NREL). Golden, CO. p. USA; 2018.

[74] Prume K., Viehweg J. (2015).

[75] U.S. Environmental Protection Agency. Method 1312: Synthetic Precipitation Leaching Procedure; SW-846 Test Methods for Evaluating Solid Wastes. www.epa.gov/hw-sw846/sw-846-test-method-1312-synthetic-precipitation-leaching-procedure; 1994.

[76] Townsend T.J., Yong-Chul Tolaymat T. A Guide to the Use of Leaching Tests in Solid Waste Management Decision Making. University of Florida. https://semspub.epa.gov/work/09/1112378.pdf; 2003.

[77] American Society for. Testing Materials. ASTM D3987-85 Standard Test Method for Shake Extraction of Solid Waste with Water. www.astm.org/d39885r04.html; 2017.

[78] Marshack J.B. (1986).

[79] Panthi G., Bajagain R., An Y.-J., Jeong S.-W. (2021). Leaching potential of chemical species from real perovskite and silicon solar cells. Process Saf. Environ. Protect..

[80] Su P., Liu Y., Zhang J., Chen C., Yang B., Zhang C., Zhao X. (2020). Pb-based perovskite solar cells and the underlying pollution behind clean energy: dynamic leaching of toxic substances from discarded perovskite solar cells. J. Phys. Chem. Lett..

[81] Li Z., Wu X., Wu S., Gao D., Dong H., Huang F., Hu X., Jen A.K.Y., Zhu Z. (2022). An effective and economical encapsulation method for trapping lead leakage in rigid and flexible perovskite photovoltaics. Nano Energy.

[82] Moody N., Sesena S., deQuilettes D.W., Dou B.D., Swartwout R., Buchman J.T., Johnson A., Eze U., Brenes R., Johnston M. (2020). Assessing the regulatory requirements of lead-based perovskite photovoltaics. Joule.

[83] Li Z., Wu X., Li B., Zhang S., Gao D., Liu Y., Li X., Zhang N., Hu X., Zhi C. (2021). Sulfonated graphene aerogels enable safe-to-use flexible perovskite solar modules. Adv. Energy Mater..

[84] Moody N. (2020).

[85] Li X., Zhang F., Wang J., Tong J., Xu T., Zhu K. (2021). On-device lead-absorbing tapes for sustainable perovskite solar cells. Nat. Sustain..

[86] Jiang Y., Qiu L., Juarez-Perez E.J., Ono L.K., Hu Z., Liu Z., Wu Z., Meng L., Wang Q., Qi Y. (2019). Reduction of lead leakage from damaged lead halide perovskite solar modules using self-healing polymer-based encapsulation. Nat. Energy.

[87] Li X., Zhang F., He H., Berry J.J., Zhu K., Xu T. (2020). On-device lead sequestration for perovskite solar cells. Nature.

[88] Niu B., Wu H., Yin J., Wang B., Wu G., Kong X., Yan B., Yao J., Li C.-Z., Chen H. (2021). Mitigating the lead leakage of high-performance perovskite solar cells via in situ polymerized networks. ACS Energy Lett..

[89] Wang C., Song Z., Li C., Zhao D., Yan Y. (2019). Low-bandgap mixed tin-lead perovskites and their applications in all-perovskite tandem solar cells. Adv. Funct. Mater..

[90] Alsalloum A.Y., Turedi B., Zheng X., Mitra S., Zhumekenov A.A., Lee K.J., Maity P., Gereige I., AlSaggaf A., Roqan I.S. (2020). Low-temperature crystallization enables 21.9% efficient single-crystal MAPbI3 inverted perovskite solar cells. ACS Energy Lett..

[91] Boopathi K.M., Karuppuswamy P., Singh A., Hanmandlu C., Lin L., Abbas S.A., Chang C.C., Wang P.C., Li G., Chu C.W. (2017). Solution-processable antimony-based light-absorbing materials beyond lead halide perovskites. J. Mater. Chem..

[92] Chen M., Ju M.-G., Garces H.F., Carl A.D., Ono L.K., Hawash Z., Zhang Y., Shen T., Qi Y., Grimm R.L. (2019). Highly stable and efficient all-inorganic lead-free perovskite solar cells with native-oxide passivation. Nat. Commun..

[93] Hu W., He X., Fang Z., Lian W., Shang Y., Li X., Zhou W., Zhang M., Chen T., Lu Y. (2020). Bulk heterojunction gifts bismuth-based lead-free perovskite solar cells with record efficiency. Nano Energy.

[94] Ito N., Kamarudin M.A., Hirotani D., Zhang Y., Shen Q., Ogomi Y., Iikubo S., Minemoto T., Yoshino K., Hayase S. (2018). Mixed Sn–Ge perovskite for enhanced perovskite solar cell performance in air. J. Phys. Chem. Lett..

[95] Jain S.M., Phuyal D., Davies M.L., Li M., Philippe B., De Castro C., Qiu Z., Kim J., Watson T., Tsoi W.C. (2018). An effective approach of vapour assisted morphological tailoring for reducing metal defect sites in lead-free, (CH3NH3)3Bi2I9 bismuth-based perovskite solar cells for improved performance and long-term stability. Nano Energy.

[96] Jeon N.J., Na H., Jung E.H., Yang T.-Y., Lee Y.G., Kim G., Shin H.-W., Il Seok S., Lee J., Seo J. (2018). A fluorene-terminated hole-transporting material for highly efficient and stable perovskite solar cells. Nat. Energy.

[97] Ji L., Zhang T., Wang Y., Liu D., Chen H., Zheng H., Peng X., Yuan S., Chen Z.D., Li S. (2022). Regulating crystallization dynamics and crystal orientation of methylammonium tin iodide enables high-efficiency lead-free perovskite solar cells. Nanoscale.

[98] Jiang X., Wang F., Wei Q., Li H., Shang Y., Zhou W., Wang C., Cheng P., Chen Q., Chen L., Ning Z. (2020). Ultra-high open-circuit voltage of tin perovskite solar cells via an electron transporting layer design. Nat. Commun..

[99] Kopacic I., Friesenbichler B., Hoefler S.F., Kunert B., Plank H., Rath T., Trimmel G. (2018). Enhanced performance of germanium halide perovskite solar cells through compositional engineering. ACS Appl. Energy Mater..

[100] Krishnamoorthy T., Ding H., Yan C., Leong W.L., Baikie T., Zhang Z., Sherburne M., Li S., Asta M., Mathews N., Mhaisalkar S.G. (2015). Lead-free germanium iodide perovskite materials for photovoltaic applications. J. Mater. Chem..

[101] Liao W., Zhao D., Yu Y., Shrestha N., Ghimire K., Grice C.R., Wang C., Xiao Y., Cimaroli A.J., Ellingson R.J. (2016). Fabrication of efficient low-bandgap perovskite solar cells by combining formamidinium tin iodide with methylammonium lead iodide. J. Am. Chem. Soc..

[102] Liu X., Wu T., Chen J.-Y., Meng X., He X., Noda T., Chen H., Yang X., Segawa H., Wang Y., Han L. (2020). Templated growth of FASnI 3 crystals for efficient tin perovskite solar cells. Energy Environ. Sci..

[103] Lyu M., Zhang M., Cooling N.A., Jiao Y., Wang Q., Yun J.-H., Vaughan B., Triani G., Evans P., Zhou X. (2016). Highly compact and uniform CH3NH3Sn0.5Pb0.5I3 films for efficient panchromatic planar perovskite solar cells. Sci. Bull..

[104] Nishimura K., Kamarudin M.A., Hirotani D., Hamada K., Shen Q., Iikubo S., Minemoto T., Yoshino K., Hayase S. (2020). Lead-free tin-halide perovskite solar cells with 13% efficiency. Nano Energy.

[105] Qiu J., Xia Y., Chen Y., Huang W. (2019). Management of crystallization kinetics for efficient and stable low-dimensional Ruddlesden–Popper (LDRP) lead-free perovskite solar cells. Adv. Sci..

[106] Singh A., Boopathi K.M., Mohapatra A., Chen Y.F., Li G., Chu C.W. (2018). Photovoltaic performance of vapor-assisted solution-processed layer polymorph of Cs3Sb2I9. ACS Appl. Mater. Interfaces.

[107] Wang K., Jin Z., Liang L., Bian H., Bai D., Wang H., Zhang J., Wang Q., Liu S. (2018). All-inorganic cesium lead iodide perovskite solar cells with stabilized efficiency beyond 15. Nat. Commun..

[108] Wang Y., Tu J., Li T., Tao C., Deng X., Li Z. (2019). Convenient preparation of CsSnI 3 quantum dots, excellent stability, and the highest performance of lead-free inorganic perovskite solar cells so far. J. Mater. Chem..

[109] Zhao Z., Gu F., Li Y., Sun W., Ye S., Rao H., Liu Z., Bian Z., Huang C. (2017). Mixed-organic-cation tin iodide for lead-free perovskite solar cells with an efficiency of 8.12. Adv. Sci..

[110] Ke W., Stoumpos C.C., Kanatzidis M.G. (2019). “Unleaded” perovskites: status quo and future prospects of tin-based perovskite solar cells. Adv. Mater..

[111] Liao W., Zhao D., Yu Y., Grice C.R., Wang C., Cimaroli A.J., Schulz P., Meng W., Zhu K., Xiong R.G., Yan Y. (2016). Lead-free inverted planar formamidinium tin triiodide perovskite solar cells achieving power conversion efficiencies up to 6.22. Adv. Mater..

[112] Tai Q., Cao J., Wang T., Yan F. (2019). Recent advances toward efficient and stable tin-based perovskite solar cells. EcoMat.

[113] Liu F.-W., Biesold G., Zhang M., Lawless R., Correa-Baena J.-P., Chueh Y.-L., Lin Z. (2021). Recycling and recovery of perovskite solar cells. Mater. Today.

[114] Chen B., Fei C., Chen S., Gu H., Xiao X., Huang J. (2021). Recycling lead and transparent conductors from perovskite solar modules. Nat. Commun..

[115] Park S.Y., Park J.-S., Kim B.J., Lee H., Walsh A., Zhu K., Kim D.H., Jung H.S. (2020). Sustainable lead management in halide perovskite solar cells. Nat. Sustain..

[116] Chen S., Deng Y., Xiao X., Xu S., Rudd P.N., Huang J. (2021). Preventing lead leakage with built-in resin layers for sustainable perovskite solar cells. Nat. Sustain..

[117] National Renewable Energy Laboratory (2021). To toss, repair, or recycle? How human behavior affects the fate of aging solar panels. Analysts Apply New Approach to Understand Consumer Decisions about Aging Modules, Identify Secondary Markets toward a Circular Economy. Sept.

[118] Libby C., Shaw S. (2019).

[119] Libby C., Shaw S., Heath G., Wambach K. (2018).

[120] First Solar. Recycling Brochure. www.firstsolar.com/-/media/First-Solar/Sustainability-Documents/Recycling/First-Solar-Recycling-Brochure.ashx; 2017.

[121] Recycle PV Solar. Establish a Recycling Program. https://recyclepv.solar/establish-a-recycling-program/; 2022.

[122] Tian X., Stranks S.D., You F. (2021). Life cycle assessment of recycling strategies for perovskite photovoltaic modules. Nat. Sustain..

