# Supplementary material for: Environmental and health risks of perovskite solar modules: Case for better test standards and risk mitigation solutions
Source: iScience. 2022 Dec 15;26(1):105807. doi: 10.1016/j.isci.2022.105807 (PMC9860350; doi:10.1016/j.isci.2022.105807)
Supplement: Document S1. Table S1 [file mmc1.pdf]

iScience, Volume *26*

Supplemental information

Environmental and health risks of perovskite solar modules: Case for better test standards and risk mitigation solutions

Christa E. Torrence, Cara S. Libby, Wanyi Nie, and Joshua S. Stein

Table S1. Perovskite PV Cell Composition and PCE Data – Related to Figure 6.

Num	Composition	Category	PCE (%)	Source	DOI
**1**	$CsPbI_{3}$	Pb	15.07	All-inorganic cesium lead iodide perovskite solar cells with stabilized efficiency beyond 15%	https://doi.org/10.1038/s41467-018-06915-6
**2**	$MAPbI_{3}$	Pb	21.9	Low-Temperature Crystallization Enables 21.9% Efficient Single-Crystal MAPbI3 Inverted Perovskite Solar Cells	https://doi.org/10.1021/acsenergylett.9b02787
**3**	$FAPbI_{3}$	Pb	25.8	Perovskite solar cells with atomically coherent interlayers on SnO2 electrodes	https://doi.org/10.1038/s41586-021-03964-8
**4**	$(FAPbI_{3})_{0.95}(MAPbBr_{3})_{0.05}$	Pb	23.2	A fluorene-terminated hole-transporting material for highly efficient and stable perovskite solar cells	https://doi.org/10.1038/s41560-018-0200-6
**5**	$MASn_{0.5}Pb_{0.5}I_{3}$	Pb/Sn	6.87	Highly compact and uniform CH3NH3Sn0.5Pb0.5I3 films for efficient panchromatic planar perovskite solar cells	https://doi.org/10.1007/s11434-016-1147-2
**6**	$MASn_{0.25}Pb_{0.75}I_{3}$	Pb/Sn	14.35	Stable Low-Bandgap Pb-Sn Binary Perovskites for Tandem Solar Cells	https://doi.org/10.1002/adma.201602696
**7**	$(FASnI_{3})_{0.6}(MAPbI_{3})_{0.4}$	Pb/Sn	15.08	Fabrication of Efficient Low-Bandgap Perovskite Solar Cells by Combining Formamidinium Tin Iodide with Methylammonium Lead Iodide	https://doi.org/10.1021/jacs.6b08337
**8**	$MASnI_{3}$	Sn	9.59	Regulating crystallization dynamics and crystal orientation of methylammonium tin iodide enables high-efficiency lead-free perovskite solar cells	https://doi.org/10.1039/D1NR06802D

**9**	$MASnIBr_{2}$	Sn	5.73	“Unleaded” Perovskites: Status Quo and Future Prospects of Tin-Based Perovskite Solar Cells	https://doi.org/10.1002/adma.201803230
**10**	$FASnI_{3}$	Sn	11.22	Templated growth of FASnI3 crystals for efficient tin perovskite solar cells	https://doi.org/10.1039/D0EE01845G
**11**	$CsSnI_{3}$	Sn	5.03	Convenient preparation of CsSnI3 quantum dots, excellent stability, and the highest performance of lead-free inorganic perovskite solar cells so far	https://doi.org/10.1039/C8TA10901J
**12**	$FA_{0.98}EDA_{0.01}SnI_{3}$	Sn	13.24	Lead-free tin-halide perovskite solar cells with 13% efficiency	https://doi.org/10.1016/j.nanoen.2020.104858
**13**	$(FA)_{0.75}(MA)_{0.25}SnI_{3}$	Sn	8.12	Mixed-Organic-Cation Tin Iodide for Lead-Free Perovskite Solar Cells with an Efficiency of 8.12%	https://doi.org/10.1002/advs.201700204
**14**	$PEA_{x}FA_{1_x}SnI_{3}$	Sn	12.4	Ultra-high open-circuit voltage of tin perovskite solar cells via an electron transporting layer design	https://doi.org/10.1038/s41467-020-15078-2
**15**	$BA_{2}MA_{3}Sn_{4}I1_{3}$	Sn	4.03	Management of Crystallization Kinetics for Efficient and Stable Low-Dimensional Ruddlesden-Popper (LDRP) Lead-Free Perovskite Solar Cells	https://doi.org/10.1002/advs.201800793
**16**	$Cs_{2}SnI_{6}$	Sn	0.96	From unstable CsSnI3 to air-stable Cs2SnI6: A lead-free perovskite solar cell light absorber with bandgap of 1.48 eV and high absorption coefficient	https://doi.org/10.1016/j.solmat.2016.09.022
**17**	$CsSn_{0.5}Ge_{0.5}I_{3}$	Sn/Ge	7.11	Highly stable and efficient all-inorganic lead-free perovskite solar cells with native-oxide passivation	https://doi.org/10.1038/s41467-018-07951-y

**18**	$FA_{0.75}MA_{0.25}Sn_{0.95}Ge_{0.05}I_{3}$	Sn/Ge	4.48	Mixed Sn-Ge Perovskite for Enhanced Perovskite Solar Cell Performance in Air	https://doi.org/10.1021/acs.jpclett.8b00275
**19**	$MAGeI_{2.7}Br_{0.3}$	Ge	0.57	Enhanced Performance of Germanium Halide Perovskite Solar Cells through Compositional Engineering	https://doi.org/10.1021/acsaem.8b00007
**20**	$CsGeI_{3}$	Ge	0.27	Lead-free germanium iodide perovskite materials for photovoltaic applications	https://doi.org/10.1039/C5TA05741H
**21**	$MAGeI_{3}$	Ge	0.2	Lead-free germanium iodide perovskite materials for photovoltaic applications	https://doi.org/10.1039/C5TA05741H
**22**	$Cs_{3}Bi_{2}I_{9}$ $Ag_{3}Bi_{2}I_{9}$	Bi	3.59	Bulk heterojunction gifts bismuth-based lead-free perovskite solar cells with record efficiency	https://doi.org/10.1016/j.nanoen.2019.104362
**23**	$Cs_{3}Bi_{2}I_{9}$	Bi	1	Bismuth Based Hybrid Perovskites A3Bi2I9 (A: Methylammonium or Cesium) for Solar Cell Application	https://doi.org/10.1002/adma.201501978
**24**	$Cs_{2}AgBiBr_{6}$	Bi	2.51	Composition Stoichiometry of Cs2AgBiBr6 Films for Highly Efficient Lead-Free Perovskite Solar Cells	https://doi.org/10.1021/acs.nanolett.9b00238
**25**	$MA_{3}Bi_{2}I_{9}$	Bi	3.17	An effective approach of vapour assisted morphological tailoring for reducing metal defect sites in lead-free, (CH3NH3)3Bi2I9 bismuth-based perovskite solar cells for improved performance and long-term stability	https://doi.org/10.1016/j.nanoen.2018.05.003
**26**	$AgBi_{2}I_{7}$	Bi	1.22	Pure Cubic-Phase Hybrid Iodobismuthates AgBi2 I7 for Thin-Film Photovoltaics	https://doi.org/10.1002/anie.201603608

**27**	$Cs_{3}Bi_{2}I_{10}$	Bi	0.4	Extended Photo-Conversion Spectrum in Low-Toxic Bismuth Halide Perovskite Solar Cell	https://doi.org/10.1021/acs.jpclett.6b01452
**28**	$Cs_{3}Sb_{2}I_{9}$	Sb	1.5	Photovoltaic Performance of Vapor-Assisted Solution-Processed Layer Polymorph of Cs3Sb2I9	https://doi.org/10.1021/acsami.7b16349
**29**	$MA_{3}Sb_{2}I_{9}$	Sb	2.04	Solution-processable antimony-based light-absorbing materials beyond lead halide perovskites	https://doi/org/10.1039/C7TA06679A

Compositions are formatted in TeX for Matplotlib.
